# Mechanistic studies in *Drosophila* and chicken give new insights into functions of DVL1 in dominant Robinow syndrome

**DOI:** 10.1242/dmm.049844

**Published:** 2023-04-13

**Authors:** Sarah J. Gignac, Katja R. MacCharles, Katherine Fu, Kywana Bonaparte, Gamze Akarsu, Thalia W. Barrett, Esther M. Verheyen, Joy M. Richman

**Affiliations:** ^1^ Life Sciences Institute and Faculty of Dentistry, University of British Columbia, Vancouver, British Columbia V6T 1Z3, Canada; ^2^ Department of Molecular Biology and Biochemistry, Centre for Cell Biology, Development and Disease, Simon Fraser University, Burnaby, British Columbia V5A 1S6, Canada

**Keywords:** Wnt signaling, Dishevelled 1, Skeletogenesis, Planar cell polarity, *Drosophila*, Chicken embryo

## Abstract

The study of rare genetic diseases provides valuable insights into human gene function. The autosomal dominant or autosomal recessive forms of Robinow syndrome are genetically heterogeneous, and the common theme is that all the mutations lie in genes in Wnt signaling pathways. Cases diagnosed with Robinow syndrome do survive to adulthood with distinct skeletal phenotypes, including limb shortening and craniofacial abnormalities. Here, we focus on mutations in dishevelled 1 (DVL1), an intracellular adaptor protein that is required for both canonical (β-catenin-dependent) or non-canonical (requiring small GTPases and JNK) Wnt signaling. We expressed human wild-type *DVL1* or *DVL1* variants alongside the endogenous genome of chicken and *Drosophila*. This design is strategically suited to test for functional differences between mutant and wild-type human proteins in relevant developmental contexts. The expression of variant forms of *DVL1* produced a major disorganization of cartilage and *Drosophila* wing morphology compared to expression of wild-type *DVL1*. Moreover, the variants caused a loss of canonical and gain of non-canonical Wnt signaling in several assays. Our data point to future therapies that might correct the levels of Wnt signaling, thus improving skeletal growth.

## Teaser

**Summary:** Mutations affecting the C-terminus of DVL1, a protein involved in Robinow syndrome, alter the balance of canonical and non-canonical Wnt signaling in the chicken limb and *Drosophila* wing.

## INTRODUCTION

Robinow Syndrome (RS) is a rare, genetically heterogeneous disorder (affecting one in 500,000 people) that is inherited as either an autosomal dominant or recessive disease. The two types can be distinguished by inheritance patterns and severity of phenotypes, with recessive forms caused by *ROR2* mutations leading to more severe clinical outcomes (Afzal et al., 2000; van Bokhoven et al., 2000). In autosomal dominant RS, mutations occur in five genes (Zhang et al., 2022) and give rise to a set of common phenotypes, including ‘fetal facies’ (hypertelorism or wide-set eyes, wide nasal bridge, midfacial hypoplasia, micrognathia, or smaller mandible and dental irregularities) (Zhang et al., 2022). The limbs (especially the forelimbs) are characteristically shorter (mesomelia or brachydactyly) (Abu-Ghname et al., 2021; Kaissi et al., 2020; Zhang et al., 2021). The mutated genes code for the WNT5A ligand (MIM: 180700) (Person et al., 2010), the frizzled 2 (FZD2) receptor (White et al., 2018) or the adaptor proteins dishevelled 1, 2 and 3 (DVL1, MIM: 616331; DVL2, MIM: 602151; DVL3, MIM: 616894) (Bunn et al., 2015; White et al., 2015, 2018, 2016; Zhang et al., 2021). The *DVL1*, *DVL2* and *DVL3* mutations cause frameshifts that substitute an abnormal basic, proline-rich C-terminal tail (Zhang et al., 2021). The human genetics studies have proposed that the autosomal dominant forms of RS, including those caused by DVL mutations create gain-of-function rather than loss-of-function proteins (Zhang et al., 2021). Support for the idea comes from the fact that heterozygous null mutations of *Wnt5a* (Yamaguchi et al., 1999), *Dvl1*, *Dvl2*, *Dvl3* (Wynshaw-Boris, 2012) and *Fzd2* (Yu et al., 2010) in mice are normal and viable. Moreover, the full germline knockouts of *Dvl1*, *Dvl2* or *Dvl3* are skeletally normal (Etheridge et al., 2008; Lijam et al., 1997; Wynshaw-Boris, 2012), suggesting that haploinsufficiency is not the mechanism underlying the RS skeletal phenotypes.

**Fig. 1.**
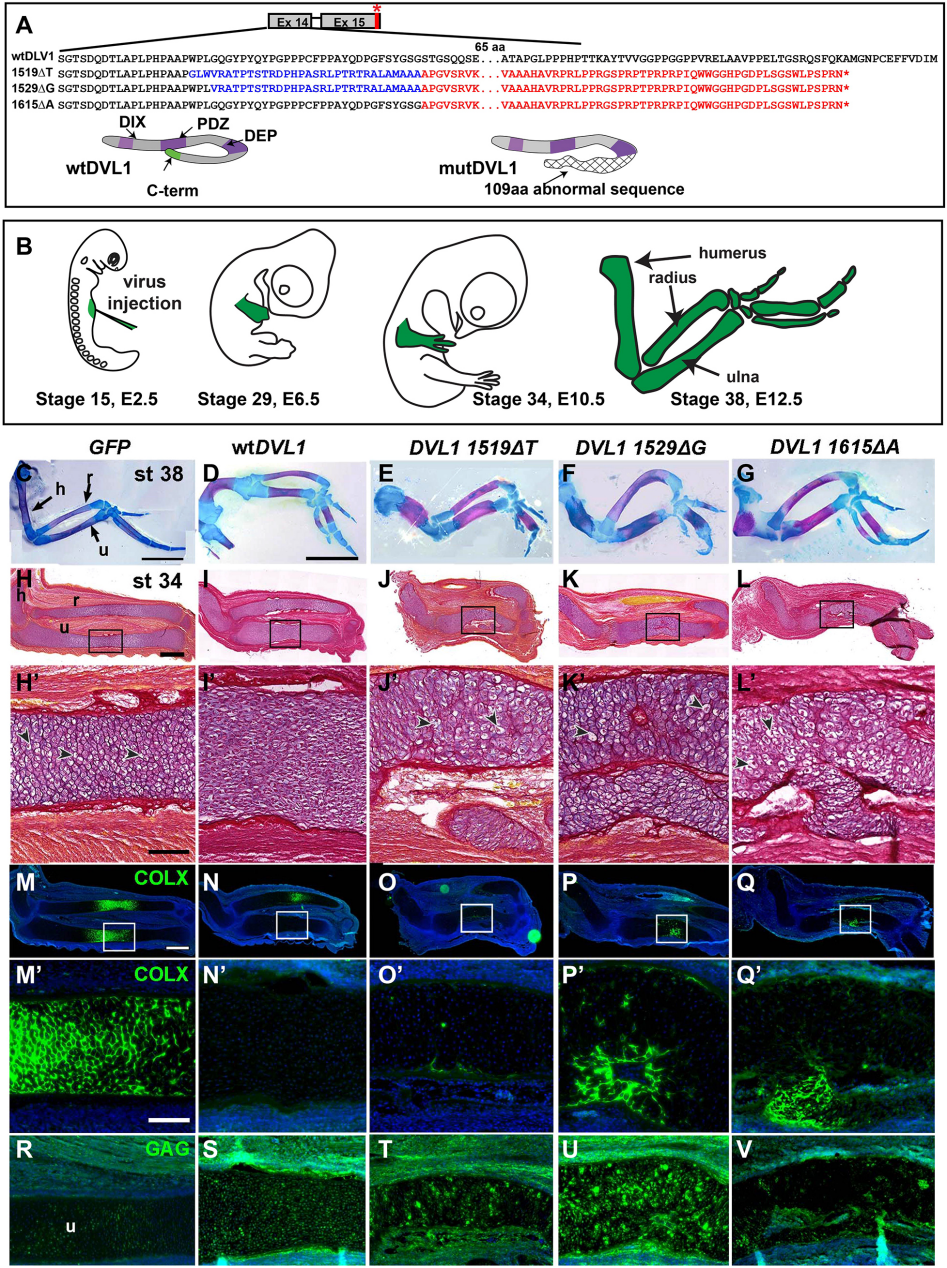
**Skeletal phenotypes obtained from misexpression of *DVL1* retroviruses in the forelimb.** (A) Sequence changes caused by the three frameshifted mutations in this study leading to an almost complete replacement of the C-terminus with a 141-amino-acid-long abnormal sequence with a new stop codon (red asterisk). (B) Stage 15 chick embryos were injected with RCAS viruses into the region of the developing forelimb and fixed at various time points. (C-G) Limbs stained in wholemount with Alcian Blue for cartilage and Alizarin Red for bone showing straight bones in *GFP-*expressing forelimbs (C). wt*DVL1*-expressing limbs had shorter and bent bones (D). The limbs injected with *DVL1^1519ΔT^* (E), *DVL1^1529ΔG^* (F) or *DVL1^1615ΔA^* (G) had shortened, thickened and bent bones. (H-L′) Sagittal sections of stage 34 limbs. (H′) In *GFP*-expressing controls, hypertrophic chondrocytes in the diaphysis were regular in shape and had small lacunae (arrowheads). (I′) Expression of wt*DVL1* made it difficult to see individual lacunae. (J-L′) The cartilage was irregular in shape and chondrocytes were disorganized (arrowheads) upon expression of *DVL1* variants. Hypertrophic cells were not regularly arranged. (M-Q′) Near-adjacent sections stained with anti-COLX antibody to mark hypertrophic chondrocytes. (M′) In GFP-expressing controls, the COLX stain overlapped with hypertrophic chondrocytes as seen in H′. (N′) COLX staining was absent in the ulna upon expression of wt*DVL1*. (O′-Q′) Variant *DVL1*-expressing limbs showed patchy COLX expression near the perichondrium. H′-Q′ are higher-magnification views of the boxes shown in H-Q. (R-V) Anti-GAG staining on near-adjacent sections shows viral expression throughout the cartilage. Images are representative of four to six specimens (see Tables S2 and S3 for *n*-values). h, humerus; r, radius; u, ulna. Scale bars: 5 mm (C,D; bar in D applies to E-G); 500 µm (H; applies to I-L); 100 µm (H′; applies to I′-L′); 500 µm (M; applies to N-Q); 100 µm (M′; applies to N′-Q′,R-V).

The −1 frameshift in each of the 14 reported *DVL1* variants replaces the DVL1 C-terminus with an abnormal peptide that is identical for 96 amino acids (White et al., 2015; Zhang et al., 2022) (Fig. 1A). The DVL1 C-terminus contains important functional domains, including an evolutionarily conserved PDZ-binding motif at the end of the protein with which it can bind intrinsically onto the endogenous PDZ domain (Lee et al., 2015; Qi et al., 2017; Wallingford and Habas, 2005; Wang and Malbon, 2012). There is also a nuclear export signal in the C-terminus (Sharma et al., 2018), which, when lost, could lead to retention of mutant DVL1 in the nucleus. Although some researchers have postulated that the abnormal C-terminus causes the phenotypes (Bunn et al., 2015; Zhang et al., 2022), it is also possible that there are abnormalities of folding that affect the adjacent, unmodified domains.

*DVL1* mutations could alter function in both canonical and non-canonical Wnt pathways. In canonical Wnt signaling, the Wnt ligand binds to the LRP-FZD co-receptors, which then recruit DVL proteins. The DIX domain in DVL proteins subsequently interacts with axin, leading to the recruitment and inhibition of a complex of proteins called the β-catenin destruction complex (Fig. S11). Thus, β-catenin can accumulate in the cytoplasm and nucleus when canonical signaling is active (Bienz, 2014; Nusse and Clevers, 2017). In contrast, Wnt binding to FZD-ROR2 heterodimers activates the JNK-planar cell polarity (PCP) pathway (Teufel and Hartmann, 2019). DVL proteins activate PCP signaling via their DEP domain (Axelrod et al., 1998; Boutros et al., 1998; Nishita et al., 2010). Not all Wnt pathways involve DVL1 (Teufel and Hartmann, 2019). These alternative pathways would not be affected in RS patients with *DVL1* mutations. For example, the receptor ROR2 can form homodimers or heterodimers with RYK but these do not signal via DVL (Stricker et al., 2017). Thus, we focused our analysis on canonical and JNK-PCP signaling.

Here, we investigated for the first time the molecular activities of RS patient mutations in *DVL1* using two distinct and complementary model systems. We used the chicken embryo limb in which skeletal development is highly conserved with humans (Tickle, 2015). Moreover, the three-dimensional (3D) skeleton and full mineralization of the endochondral bone can only be studied *in vivo*. The chicken embryo limb bud is very accessible to manipulations ranging from local transgenesis with avian retroviruses to bead implants (Davey et al., 2018). Although human transgenes can be targeted to the limb of chickens using viruses (Gignac et al., 2019), the chicken is not a genetic model. To precisely control the location, stage of development and level of expression of a transgene, a genetic model such as *Drosophila melanogaster* is necessary*.* Indeed, the homologous gene, *dishevelled* (*dsh*), was first identified in *Drosophila*, so named because the mutations caused wing hairs to be randomly oriented (Fahmy and Fahmy, 1959). Many Wnt components were identified from genetic screens, and *Drosophila* was instrumental for working out the signaling hierarchy and interactions of these pathways (Baker, 1987; Jenny et al., 2003; Nüsslein-Volhard and Wieschaus, 1980; Nüsslein-Volhard et al., 1984; Peifer et al., 1994; Vinson and Adler, 1987; Yanagawa et al., 2002). The strategy for both animal models in this study was to express the human genes in defined regions of the developing organism, alongside the endogenous wild-type alleles. This design is particularly advantageous for studying autosomal dominant mutations that are thought to cause an interference between the mutant and wild-type protein. Indeed, our previous work on an autosomal dominant *WNT5A* point mutation found evidence for dominant interference that altered bone shape as well as partial loss of function of WNT5A due to reduction in secretion (Gignac et al., 2019; Hosseini-Farahabadi et al., 2017).

In this study, we found that hypertrophic chondrocytes were poorly organized in the chicken skeleton infected with human *DVL1* variants. These effects caused by variant *DVL1* were distinct from those of wild-type (wt) *DVL1* and those we reported for *WNT5A^C83S^* (Gignac et al., 2019). We carried out complementary experiments in *Drosophila* and found that the wing hairs were randomized more frequently following expression of *DVL1* variants, suggesting that the PCP mechanisms were disturbed. Luciferase reporter assays in HEK293 cells combined with *in vivo* readouts of canonical and JNK signaling in the fly showed a decrease in the levels of canonical signaling caused only by the mutant forms of DVL1. On the other hand, the level of the JNK-PCP branch of Wnt signaling was generally increased, particularly in *Drosophila* wing imaginal discs. These data show that RS is mediated by a complex set of molecular defects in two Wnt signaling pathways.

## RESULTS

We examined the effects of human *DVL1* mutations in chicken embryos and developing fly tissues. Our goal was to introduce the wild-type *DVL1* using virus infection in the chicken or by transgenesis in the fly and compare the effects to the variant forms and controls. By carrying out these studies in a wild-type, endogenous *DVL1*/*dsh* background, we could model the interaction between wild-type and mutant proteins, which is a crucial aspect of the human autosomal dominant RS genetics. One outcome could be dominant interference that disrupts the normal function of endogenous DVL1/Dsh. It is also possible that the mutations produce a gain of function and, in this case, variants would resemble wt*DVL1*/*dsh* overexpression. Another possibility is that the mutant protein may have novel functions and phenotypes that would be distinct from those of GFP controls or wtDVL1. Finally, if the variants were completely inactive, then no phenotypes would be produced.

### *DVL1* variants affect endochondral bone formation in the limb differently than wt*DVL1*

Viruses were injected into chicken embryos and the *in vivo* viral expression of the human *DVL1* transgene was measured using quantitative real-time PCR (qRT-PCR) (Fig. S1A). We found that all viruses were expressed and the *DVL1^1529ΔG^* variant was more highly expressed than wt*DVL1.* The other variants were expressed at comparable levels to wt*DVL1* and their expression levels were not significantly different. The addition of human *DVL1* genes in the chicken limb did not affect levels of *Gallus DVL1* (Fig. S1B).

To determine the endpoint of the phenotype, chicken embryos were injected at stage 15 or 20 [embryonic day (E) 2.5 or 3.5] (Hamburger and Hamilton, 1951, 1992) and incubated until stage 38, which showed the 3D skeletal phenotypes (Fig. 1B). Examination of cleared skeletal bones showed that there was normal development in GFP controls (Fig. 1C). The injection of viruses containing wt*DVL1* or *DVL1^1519ΔT^* at stage 20 resulted in 50% of phenotypes affecting the humerus only, which is specified later than the more distal elements (Table S1B). In contrast, more than 50% of embryos injected at stage 15 with variant or wt*DVL1* viruses had skeletal defects in the radius, ulna and humerus (Fig. 1C-G; Table S1A). However, the correlation between the phenotype and location of the virus cannot be done with the technique of whole-mount skeletal staining.

To trace the onset of the phenotypes and for a more detailed characterization, we collected other groups of embryos at Hamburger–Hamilton stages 28 (E5.5), 29 (E6.5) and 34 (E10.5) (Fig. 1H-V; Table S2). We first examined viral expression [using an antibody against group-specific antigen (GAG), a marker of viral expression] and found some variability across specimens due to initial targeting of the lateral plate mesoderm (Table S2; 27/32 had GAG staining in the bone primordia at stage 29). At stage 34, before ossification begins, some specimens lacked GAG in certain skeletal elements. We selected the subset with robust GAG staining for further structural and quantitative analysis.

### *DVL1* variants cause cartilage dysplasia and disrupt muscle patterning

**Fig. 2.**
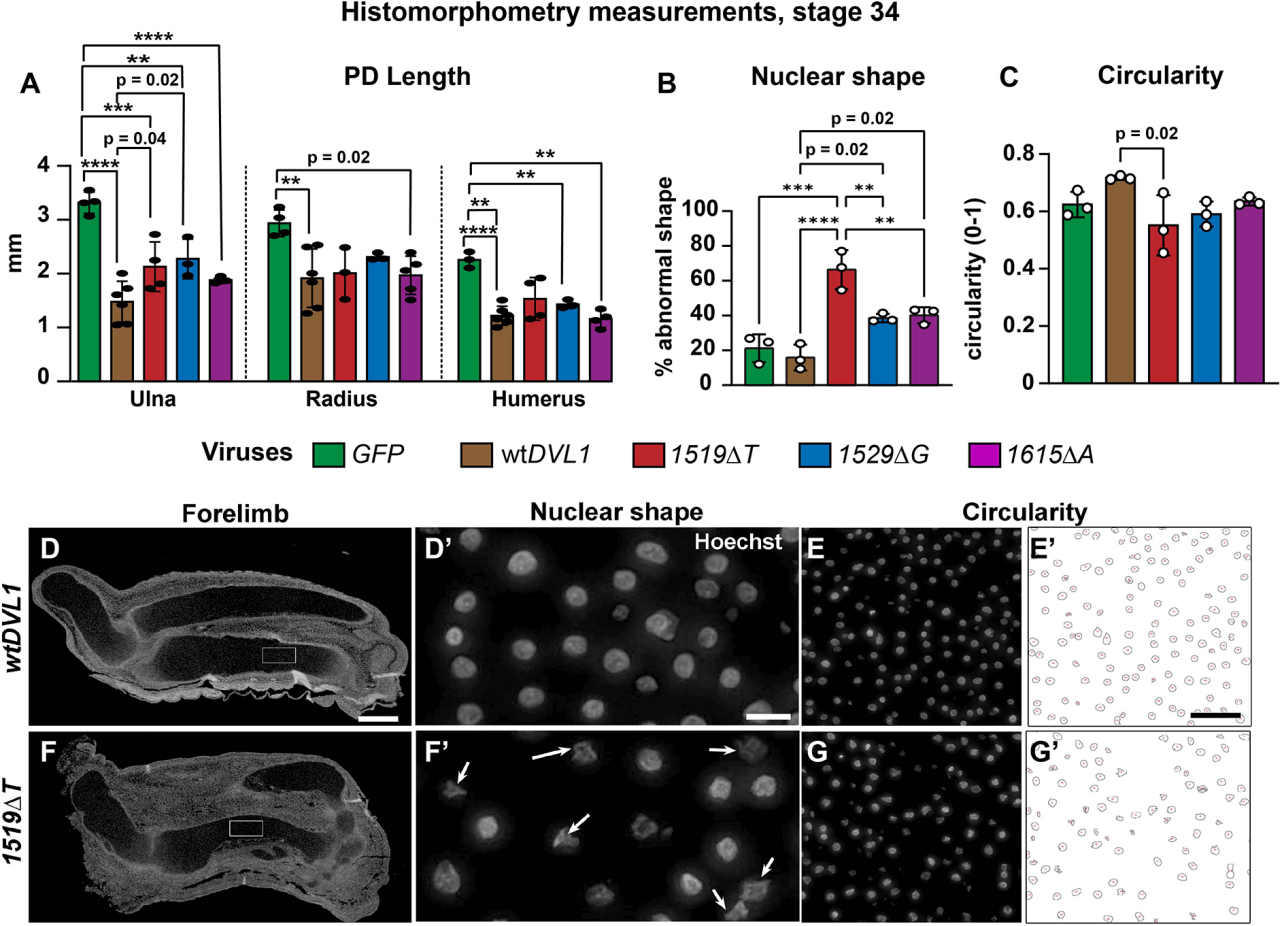
**Quantification of cartilage phenotypes at stage 34.** (A) wt*DVL1* and all *DVL1* variants similarly reduced bone length compared to *GFP* controls. Measurements in the perpendicular axis show significant increase in thickness caused by the wt*DVL1* virus in the ulna and humerus. PD, proximo-distal. (B) Percentage of abnormal nuclear shapes were significantly increased by the three variants, most of all by the *DVL1^1519ΔT^* variant. Numbers of cells counted per replicate averaged 155±48. (C) ImageJ analysis of circularity shows a similar trend where the cells expressing the *DVL1^1519ΔT^* variant are significantly less circular than those expressing wt*DVL1*. Data show the mean±s.d. One-way ANOVA and Tukey's post hoc test was used as per methods. (D,D′) Nuclei from the diaphysis appear rounded. (E,E′) Thresholding in ImageJ, followed by outlining objects. (F,F′) The *DVL1^1519ΔT^* variant caused many nuclei to lose the round shape (arrows). (G,G′) Circularity analysis in ImageJ. D′,F′ are higher-magnification views of the boxes shown in D,F. Scale bars: 200 µm (D,F); 10 µm (D′,F′); 40 µm (E,E′,G,G′). **P<0.01; ***P<0.001; ****P<0.0001.

GAG staining confirmed where the viruses had spread in relation to skeletal phenotypes (Fig. 1R-V). GFP control limbs had normal endochondral ossification initiating in the central diaphysis (Fig. 1H,H′) (Gignac et al., 2019; Vortkamp et al., 1996; Hartmann and Tabin, 2000). In contrast, the chondrocytes in the wt*DVL1* limbs had not started to hypertrophy and lacunae were not visible (Fig. 1I,I′). Variant-infected limbs had dysplastic cartilage morphology with disorganized chondrocytes and irregular borders that were not seen in the wt*DVL1*-infected limbs (Fig. 1J-L′; Fig. S2F-H′, Fig. S3A-F′). Type X collagen (COLX or COL10A1), a marker of chondrocyte hypertrophy, was strongly expressed in the diaphysis of GFP controls (Fig. 1M,M′). However in the wt*DVL1-*infected limbs, there was an absence of COLX staining in 5 out of 6 specimens (Fig. 1N,N′; Table S3). In all variant-infected limbs, small areas of the disorganized chondrocytes expressed COLX (Fig. 1O-Q′; patchy COLX staining, Table S3). As bone elongation depends on organized stacking of chondrocytes and hypertrophy, we measured the length of the skeletal elements. All bones infected with wt*DVL1* or the variants were shorter in the proximo-distal axis than those of the GFP controls (Fig. 2A).

We noticed an unusual nuclear effect in the Hoechst 33258-stained sections. The proportion of nuclei with abnormal shapes (polygonal, star-shaped or rectangular) was significantly higher in the variant-infected limbs compared to w*tDVL1*-infected limbs (Fig. 2B,D,D′,F,F′). We also used a computer-based approach to measure circularity of the nuclei (ImageJ) (Fig. 2C,E,E′,G,G′). These analyses were less sensitive but confirmed that the *DVL1^1519ΔT^*-infected chondrocytes had significantly fewer circular nuclei compared to the other conditions (Fig. 2C). Thus, the appearance of irregularly shaped chondrocyte lacunae is connected to abnormal nuclear shape and could reflect abnormal stresses in the cytoskeleton caused by the variants (Kalukula et al., 2022; Long and Lammerding, 2021).

The large disruption to chondrogenesis caused by the variants may also have affected myogenesis. The somites (demomyotome) give rise to muscle cells and these migrate into the early limb bud, mixing with the lateral plate-derived mesoderm. The timing of virus injection would coincide with muscle patterning. In controls, we found clearly delineated muscles surrounded by a myofascial sheath using an antibody against muscle actin (*n*=6; Fig. S4A-B′). Similar muscle delineation was seen in the w*tDVL1*-injected limbs, although muscles were shorter, matching the shorter radius, ulna and humerus (*n*=5; Fig. S4C-D′). In contrast, it was more difficult to find the defined muscles (Fig. S4E-J′) in the variant-infected limbs (*n*=11). The muscle cells were not always aligned with the long axis of the limb (Fig. S4E,F,F′). Thus, all the *DVL1* variants interfered with normal muscle patterning. Although there was some variability between the extent of cartilage and muscle dysplasia induced by the variants, there were distinctly different responses compared to infection by the wt*DVL1* virus.

### Cartilage dysplasia originates in the anlagen stage

**Fig. 3.**
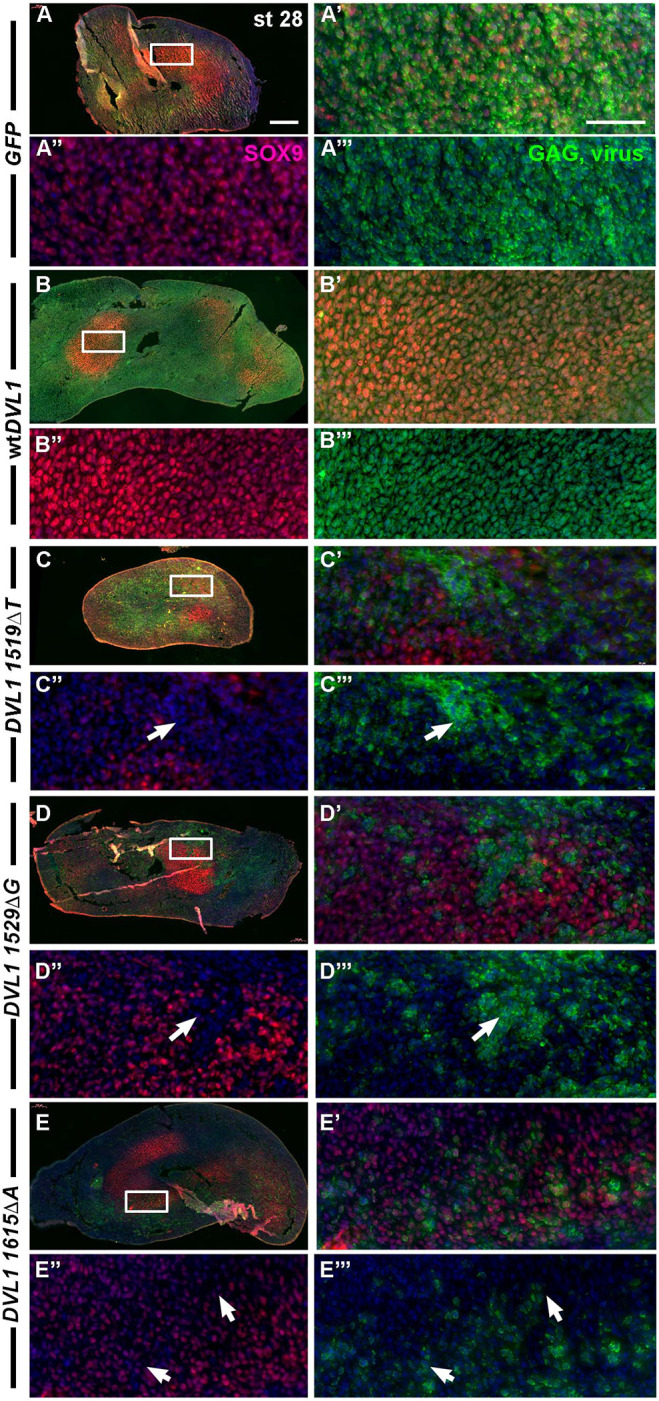
**Inhibition of chondrogenesis by *DVL1* variants contributes to skeletal dysplasia.** Sagittal sections of forelimbs injected at stage 15 and fixed 72 h post injection at stage 28. The distal tip where digits form is on the right side. (A-A‴) Chondrocytes express SOX9 (magenta) in the cartilage condensations, which overlaps with viral GAG staining (green) (*n*=3). (B-B‴) Full overlap between the wt*DVL1* virus and differentiating chondrocytes expressing SOX9 (*n*=4). (C-C‴) Some areas of the *DVL1^1519ΔT^*-infected cartilage have GAG staining, but cells are undifferentiated as shown by the absence of SOX9 staining (white arrows; *n*=5). (D-E‴) *DVL1^1529ΔG^* and *DVL1^1615ΔA^* variants (*n*=6 each) have pockets of undifferentiated cells within the cartilage elements (white arrows). Scale bars: 200 µm (A-E); 50 µm (A′-E‴).

To understand the origins of the cartilage dysplasia, we looked at earlier stages during which the cartilage elements were condensing (stages 28 and 29; 72-96 h post infection). At stage 28, the chondrocytes were strongly labeled with SOX9, a marker of chondrocyte lineage (Fig. 3A-A‴), and the staining overlapped completely with GAG staining. Similarly, in wt*DVL1*-infected limbs, cells that were SOX9-positive also showed GAG staining (Fig. 3B-B‴). However, in the variant-infected limbs, we often observed complementary GAG and SOX9 staining (Fig. 3C-E‴, white arrows). The variegated expression of the GAG staining correlates with the variable RNA expression levels of the mutant viruses measured in the same-stage limb buds (Fig. S1). At stage 29, the mottled appearance was similar to that of stage 28 (Fig. S5; Table S4).

We next checked whether the cartilage phenotypes seen at stage 34 were due to uneven patterns of cell proliferation that occurred during earlier stages of establishing the shape of the bone. As shown previously, the GFP controls in stage 29 limbs had relatively higher proliferation in the epiphyses (Fig. S6A,A′) (Gignac et al., 2019). However, in wt*DVL1* virus-injected limbs, proliferating cells were distributed throughout the cartilage element including the diaphysis (Fig. S6B,B′). All *DVL1* variant-infected limbs showed irregular borders around the developing ulna, diaphysis or humerus. There were proliferating cells throughout the cartilage element (Fig. S6C-E′) and the percentages of bromodeoxyuridine (BrdU)-positive cells were similar for all virus types (Fig. S6F). Importantly, there was no evidence of decreased proliferation in areas where the cartilage was dysplastic. We found normal domains of apoptosis along the anterior and posterior limb margins and between the digits at stage 29 (Fig. S7A-A″), and no evidence of increased numbers of apoptotic cells present in the *DVL1*-infected limbs (Fig. S7B-E″). This makes it unlikely that proliferation or apoptosis are mechanisms for the cartilage and muscle dysplasia seen at stage 34.

### wt*DVL1* and the variants randomize cell polarity

**Fig. 4.**
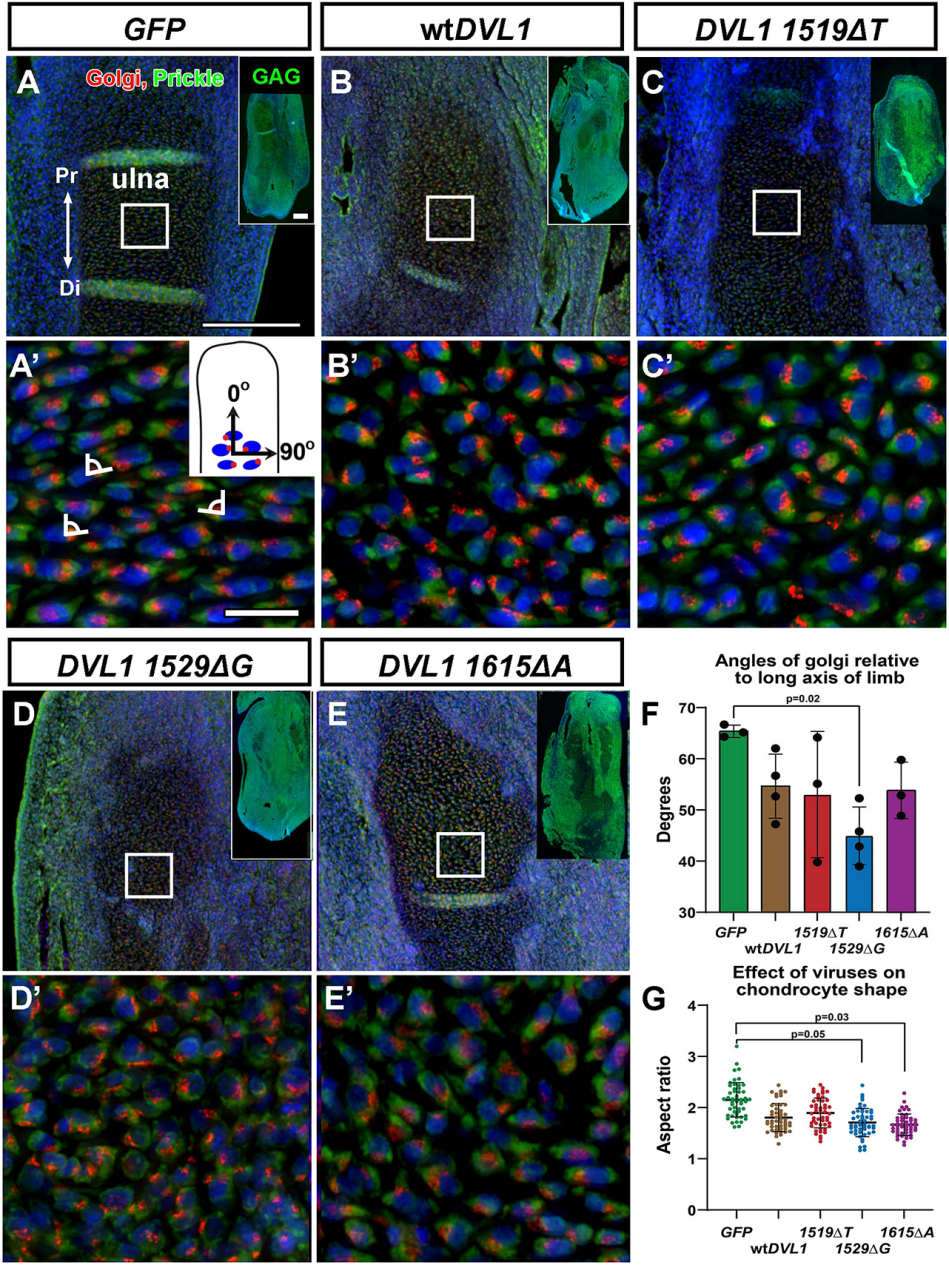
**Measurement of chondrocyte orientation and shape.** (A-E) Sagittal slices of injected forelimbs. The angle between the Golgi-nucleus axis and the long axis of the bones in the ulna was measured (between 60 and 120 cells per biological replicate). Insets in A-E show GAG staining in near-adjacent sections. A′-E′ are high-magnification views of the boxes in A-E. Pr, proximal; Di, distal. Scale bars: 200 µm (A; applies to A-E); 200 µm (inset in A; applies to insets in A-E); 20 µm (A′; applies to A′-E′). (F) Graphical representation of angular data for each virus type. The cells were significantly more randomly oriented for *DVL1^1529ΔG^* compared to *GFP* control but there was no difference between the variants and wt*DVL1*. (G) The aspect ratio (width to height) for chondrocytes was measured. The trend for the wt*DVL1* and variants was to have more rounded shapes compared to *GFP-*expressing controls. However only the cells expressing the *DVL1^1529ΔG^* and *DVL1^1615ΔA^* variants were significantly rounder than the *GFP-*expressing controls. There was no difference between the variants and wt*DVL1.* Data show the mean±s.d. *n*=3 for *GFP*, *DVL1^1519ΔT^* and *DVL1^1615ΔA^*; *n*= 4 for wt*DVL1* and *DVL1^1529ΔG^*. One-way ANOVA and Tukey's post hoc test was used for statistical analysis.

We suspected based on the dysplastic cartilage that chondrocytes had lost their typical planar polarity relative to the long axis of the limb. In studies with the *WNT5A^C83S^* variant, we found a loss of chondrocyte polarity in the limb and face (Gignac et al., 2019; Hosseini-Farahabadi et al., 2017), although the cartilages were not dysplastic. To assess polarity effects of the *DVL1* viruses, we stained for the Golgi apparatus in stage 29 cartilages, as stacking was being established. In a highly polarized cell, the Golgi bodies are located at the furthest extension of the cell. Calculation of the angle of the Golgi apparatus relative to the long axis of the limb is a measure of cell orientation. We also stained for Prickle with an antibody that recognizes both Prickle 1 and Prickle 2. Prickle is one of the key asymmetrically localized cytoplasmic components in PCP signaling (Butler and Wallingford, 2017). However, in chondrocytes, Prickle is typically expressed symmetrically at opposite ends of the flattened, polarized cells in the mouse embryo limb skeleton (Kuss et al., 2014) and chicken limb (Gignac et al., 2019). In this study, the Golgi-nucleus axes in *GFP*-injected limbs had orientations of around 65° relative to the long axis of the limb (Fig. 4A,A′,F). In wt*DVL1-* and variant*-*infected limbs, the location of the Golgi apparatus was more variable as shown by the large standard deviation (Fig. 4B-F); however, only the *DVL1^1529ΔG-^*injected limbs showed significantly different angles compared to those of the *GFP-*infected controls (Fig. 4F). The important finding is that wt*DVL1* also disrupted polarity, suggesting that any increase in DVL1 expression can affect the PCP pathway.

Prickle expression outlined the chondrocytes very clearly; therefore, we measured the effect of the various forms of *DVL1* on shape (Fig. 4G). Flattened morphology is an important prerequisite for stacking of chondrocytes and elongation of the growth plate (Li and Dudley, 2009; Li et al., 2017). The aspect ratio is the ratio of the major and minor axes and was generally close to 2 for most cells in the *GFP* (mean=2.153), wt*DVL1* (mean=1.806) and the *DVL1^1519ΔT^* (mean=1.895)-infected samples. The ratios were significantly lower for *DVL1^1529ΔG^* (1.709, *P*=0.05) and *DVL1^1615ΔA^* (1.665, *P*=0.03)-infected samples (Fig. 4G). Thus, in these chondrocyte morphology assays carried out at stage 29, we saw that increased levels of either wt or variant *DVL1* caused some loss of polarity as measured by the Golgi-nucleus angle relative to the long axis of the limb and slightly increased cell roundness. Overall, the variants did not produce differences relative to wt*DVL1* in stage 29 limbs in all the assays (proliferation, Golgi-nucleus angle, roundness and apoptosis). These early-stage results on the polarity of chondrocytes (likely mediated by abnormal PCP signaling) link to the abnormal hypertrophy and ultimately to the shortening of all bones by all forms of the *DVL1* virus at stage 34.

### DVL1 variant proteins show increased nuclear localization compared to wtDVL1

**Fig. 5.**
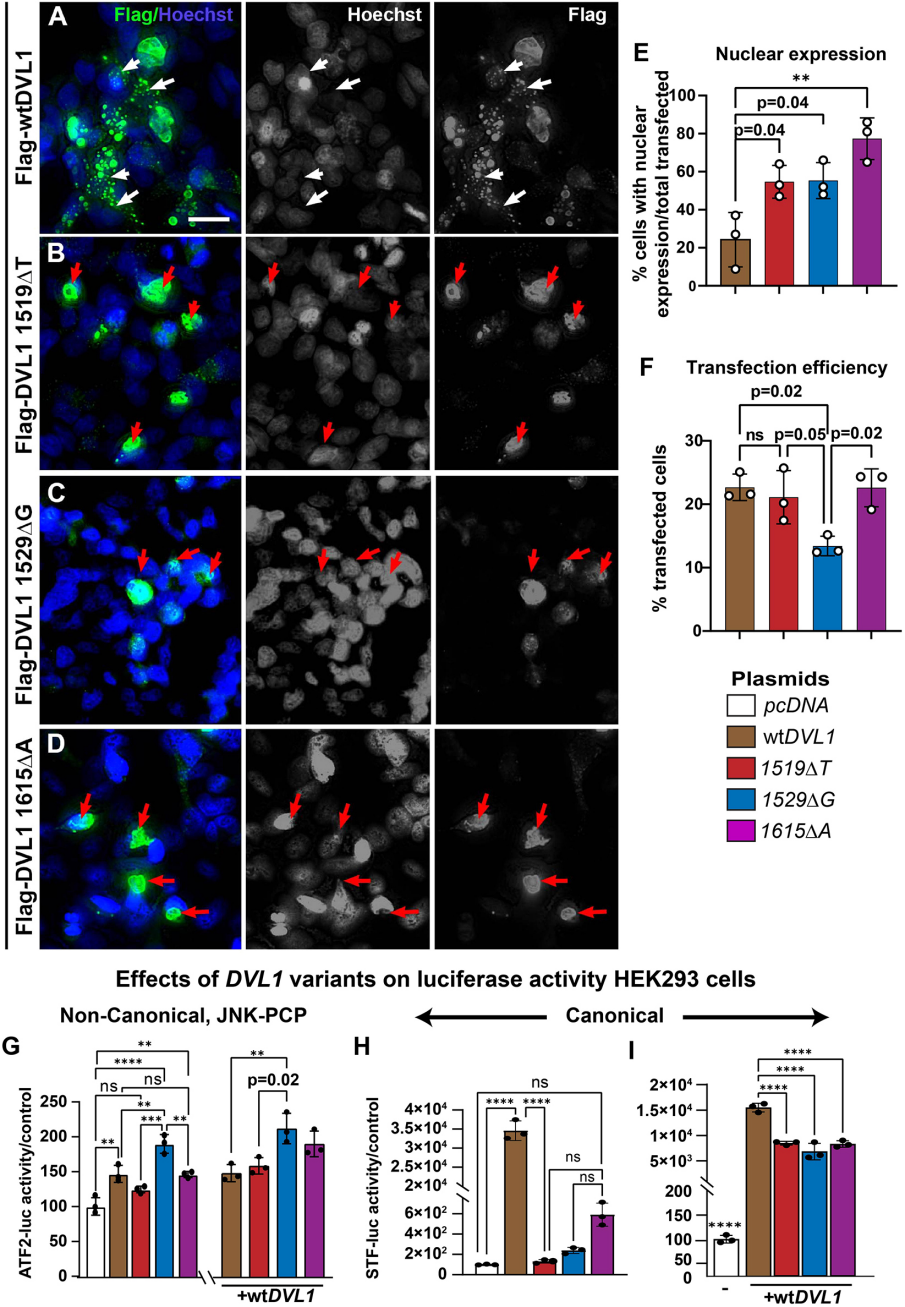
**Expression of Flag-DVL1 protein in HEK293 cells and effects on Wnt pathway reporter assays.** Cells were transfected with Flag-tagged DVL1 constructs (*n*=3 coverslips or biological replicates per virus type). (A) Flag-wtDVL1 was distributed in puncta throughout the cytoplasm (green dots, white arrows). Scale bar: 20 µm (A; applies to all panels). (B-E) Flag-tagged DVL1 variants localized more to the nucleus (red arrows), which was quantified in E. (F) Transfection efficiency was lower for the *DVL1^1529ΔG^* variant. (G) The ATF2 reporter measures JNK-PCP activity. Bars were normalized to the empty parent plasmid. Adding 50% wt*DVL1* plasmid to each transfection (right) did not lead to a significant increase in overall expression except for the *DVL1^1529ΔG^* plasmid. (H) Canonical SuperTopFlash (STF) luciferase activity was strongly induced by wt*DVL1* but not by the variants. (I) Variants in combination with equimolar amounts of wt*DVL1* significantly reduced the level of activation of STF luciferase. For G,I, 0.03 µg of the empty or wt*DVL1* plasmid was transfected with 0.03 µg of the *DVL1* variant plasmid. Data show the mean±s.d. One-way ANOVA with Tukey's post hoc test was used for all multiple comparisons. ns, not significant; **P≤0.01; ***P≤0.001; ****P≤0.0001.

As the variants lack a putative nuclear export signal, we hypothesized that there would be differences in subcellular protein localization. In N-terminally tagged Flag-wtDVL1-transfected HEK293 cells, 78% of transfected cells had numerous puncta in the cytoplasm (Fig. 5A) and 22% of cells had expression concentrated in the nucleus (Fig. 5E). In contrast, the three variant proteins were significantly more likely to be concentrated in the nucleus (Fig. 5B-E). *DVL1^1529ΔG^* variant plasmid had lower transfection efficiency (Fig. 5F) but the protein variant showed the same nuclear localization (Fig. 5C). Another study noted that a *DVL1^1519ΔT^* RS variant with an N-terminal GFP tag (Bunn et al., 2015) localized to the nucleus; however, no quantification of subcellular distribution was done in that study. The nuclear translocation in our study correlates with increased likelihood of nuclear deformation seen *in vivo* in the chondrocytes within variant-infected cartilage at stage 34.

### *DVL1* variants show dominant-negative effects on canonical signaling mediated by wt*DVL1*

We tested the activity of DVL1 proteins activity using the JNK-PCP activity reporter activating transcription factor 2 (ATF2)-luciferase (Ohkawara and Niehrs, 2011) and SuperTopFlash luciferase assays, which report β-catenin/TCF-driven transcription (Ishitani et al., 2003). The ATF2 reporter was significantly activated by infection of plasmids expressing wtDVL1 and the DVL1^1529ΔG^ and DVL1^1615ΔA^ variants compared to infection of parent plasmid controls (Fig. 5G). DVL1^1529ΔG^ significantly activated JNK signaling compared to wtDVL1 (*P*=0.02). When we combined equimolar amounts of plasmids expressing wt*DVL1* and each of the variants, there was no evidence of synergism or interference (Fig. 5G). There was also no evidence of loss of function in the JNK pathway by any of the variants.

The SuperTopFlash reporter is highly sensitive in luciferase assays compared to the ATF2 reporter in our hands (Geetha-Loganathan et al., 2014; Gignac et al., 2019; Hosseini-Farahabadi et al., 2017). As expected, the wtDVL1 protein was able to strongly activate the canonical pathway (Fig. 5H), whereas DVL1 variants were poor activators (Fig. 5H). When plasmids expressing the variants were added in equimolar concentration with the wt*DVL1* plasmid, all three had significant dominant-negative effects on wtDVL1 (Fig. 5I). Thus, we have a complex pattern of elevated JNK activity with some variants and a clear reduction in canonical signaling caused by the three variant forms of DVL1 within the same human cell type. The balance of signaling between Wnt pathways appears to be disrupted, which may contribute to RS pathogenesis.

### Expression of human *DVL1* mutant genes in *Drosophila*

We next used assays in *Drosophila* to probe the *in vivo* effects of *DVL1* variants on the polarity of adult wing hairs, which is a robust readout of PCP signaling (Adler, 2012). Numerous genetically encoded reporters enabled us to determine levels of canonical and non-canonical signaling *in vivo* in developing wing imaginal discs.

In these assays, we used the *Gal4-UAS* system to control spatial and temporal transgene expression. Furthermore, *Gal4*-driven expression is temperature dependent, so higher levels of expression are induced at 29°C compared to 25°C (Duffy, 2002). The *DVL1* transgenes were inserted into the same attp40 chromosomal site to allow for comparable expression levels. To minimize effects on the overall health of the organism, we used targeted expression to generate viable animals with subsets of mutant tissues (similar to the chicken).

Transgenes were expressed using the *dpp-Gal4* driver in larval salivary glands, and qRT-PCR analyses showed that all transgenes were expressed with comparable mRNA levels both at 25°C and 29°C (Fig. S8A). Western blotting showed that both the wtDVL1 and variant proteins were expressed at the expected size of 85 kDa (Fig. S8B). However, the expression of wtDVL1 and DVL1^1615ΔA^ proteins at 25°C was consistently lower than that of the other variants, even though mRNA levels were comparable (Fig. S8B,C). Growing flies at 29°C increased expression of the *DVL1^1615ΔA^* transgene and resulted in levels of protein equivalent to those seen for the other variant transgenes at 25°C (Fig. S8C).

Immunofluorescence staining was performed to observe Flag-tagged DVL1 subcellular localization (Fig. S9). Egg-chamber somatic follicle cells were examined because the columnar epithelial cells are larger than in imaginal wing discs and they have apico-basal polarity unlike salivary gland cells, making it easier and more informative to see subcellular localization. When expressed using *traffic jam* (*tj*)*-Gal4*, all the DVL1 proteins formed puncta that were cytoplasmic as well as membrane associated (Fig. S9B-E′). Next, we compared the intracellular distribution of the human proteins when overexpressed with C-terminally myc-tagged *Drosophila* Dsh (Fig. S9F-J). We saw cytoplasmic puncta of Dsh-myc, similar to those reported for Dsh-GFP in a human cell line (Tree et al., 2002) and endogenous Dsh in *Drosophila* embryos (Yanagawa et al., 1995). Colocalization of both Dsh and DVL1 proteins in some puncta suggested that they may interact (Fig. S6F-J). The lack of nuclear expression of Flag-tagged human DVL1 variants in the *Drosophila* cells compared to predominantly nuclear expression in HEK293 cells may be due to differences in the cellular context.

### Human *DVL1* genes induce PCP defects in wing hairs

**Fig. 6.**
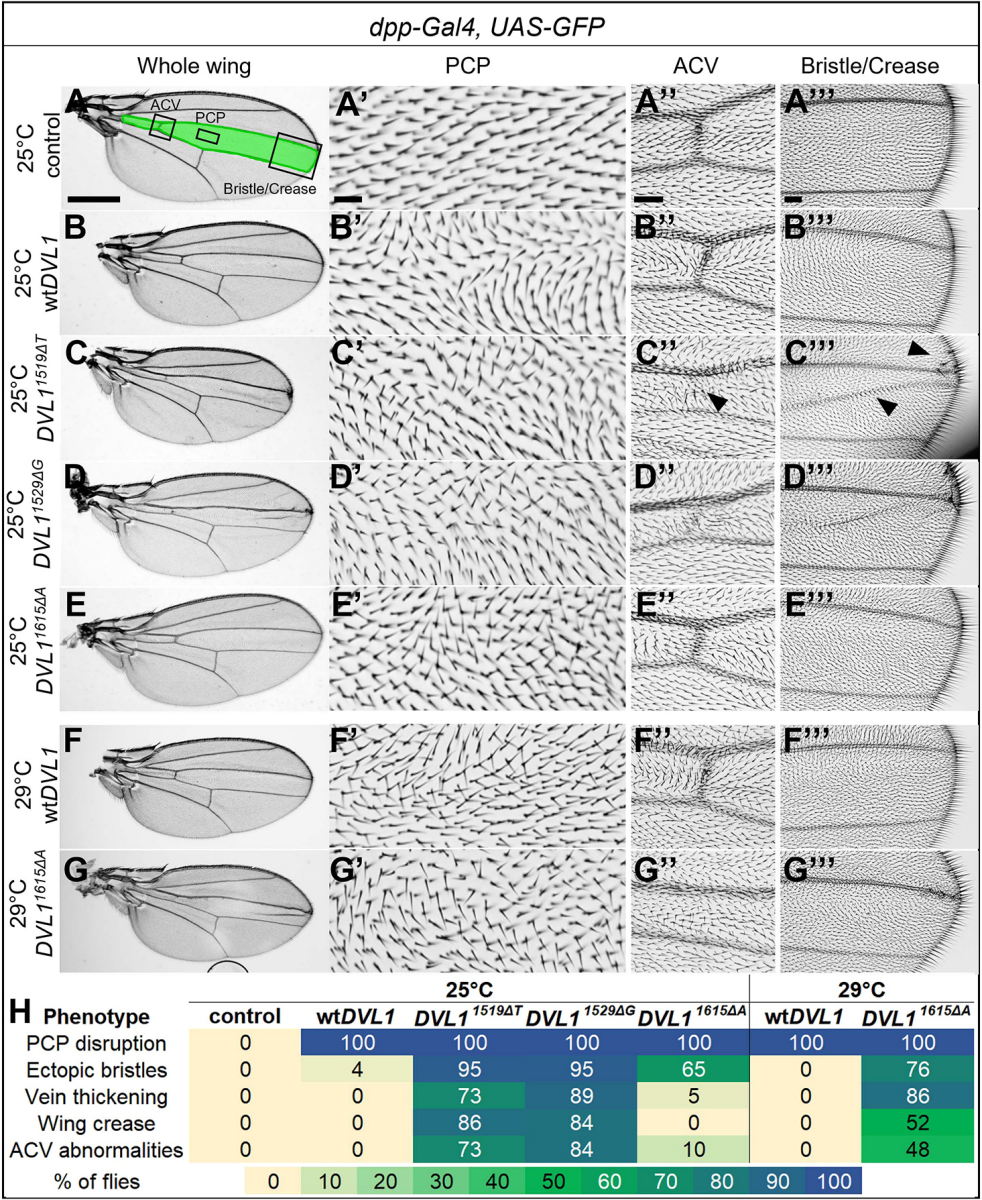
**DVL1 induces PCP defects and DVL1 variants induce neomorphic phenotypes in *Drosophila*.** (A) Control wild-type wing with shaded *dpp*-*Gal4* expression domain (green) and black boxes that correspond to magnified views presented in panels A′-A‴. (B-G) Representative *dpp>DVL1*-expressing adult female wings at 25°C or 29°C as indicated (*n*=20 or more per genotype across two experiments). (A′-G‴) Magnified views of PCP defects within a fixed region above the posterior cross vein for control. The arrowhead in C″ points to loss of the anterior cross vein (ACV). Arrowheads in C‴ indicate crease and ectopic bristles. (H) Percentage of flies with each phenotype. The actual percentage is in each cell and a colorized heat map is overlaid for clarity. Scale bars: 500 μm (A-G); 20 μm (A′-G′); 50 μm (A″-G″); 100 μm (A‴-G‴).

Adult *Drosophila* wings are covered in distally pointing parallel hairs that are oriented via the PCP pathway (Simons and Mlodzik, 2008; Wong and Adler, 1993). Detection of altered hair polarity is an established indicator of larval and pupal PCP pathway disruption. *dpp-Gal4* was used to induce expression in the wing imaginal disc along the anterior/posterior boundary (Basler and Struhl, 1994). This corresponds to the middle of the adult wing between the L3 and L4 longitudinal veins (Fig. 6A, green). Wing hair alignment in this domain can be compared to the neighboring compartments where the transgenes are not expressed. We found that all transgenes caused frequent PCP defects, seen as misaligned hair, in 100% of the wings scored (Fig. 6B′-G′,H).

### *DVL1* variants induce neomorphic phenotypes in the fly wing

While scoring the PCP phenotype, we observed that *DVL1* variant-expressing wings displayed additional, neomorphic phenotypes: vein thickening, abnormalities or absence of the anterior cross vein (Fig. 6C″-G″,H), and ectopic bristles at the edge of L3 and a wing crease between L3 and L4 veins (Fig. 6A‴-G‴,H). Although 100% of wt*DVL1*-expressing wings showed the PCP phenotype, virtually none displayed these additional phenotypes (Fig. 6H). The anterior cross vein, vein and crease phenotypes were exclusive to the *DVL1* variant-expressing wings (Fig. 6H) and the extra bristle phenotype was observed in 1/25 of wt*DVL1*-expressing wings and to variable degrees in variant wings (Fig. 6H). At 25°C, the mutant phenotypes were seen with higher penetrance in *dpp>DVL1^1519ΔT^* and *dpp>DVL1^1529ΔG^* wings compared to *dpp>DVL1^1615ΔA^* wings; however, comparable frequencies were observed in *dpp>DVL1^1615ΔA^* wings from crosses grown at 29°C, in which transgene expression is elevated (Fig. 6B-G). As the *dpp>*wt*DVL1* wings did not display these mutant phenotypes, even at 29°C, they appear to be novel effects of the variants (Fig. 6H).

### *DVL1* variants induce ectopic JNK signaling in fly wing imaginal discs

**Fig. 7.**
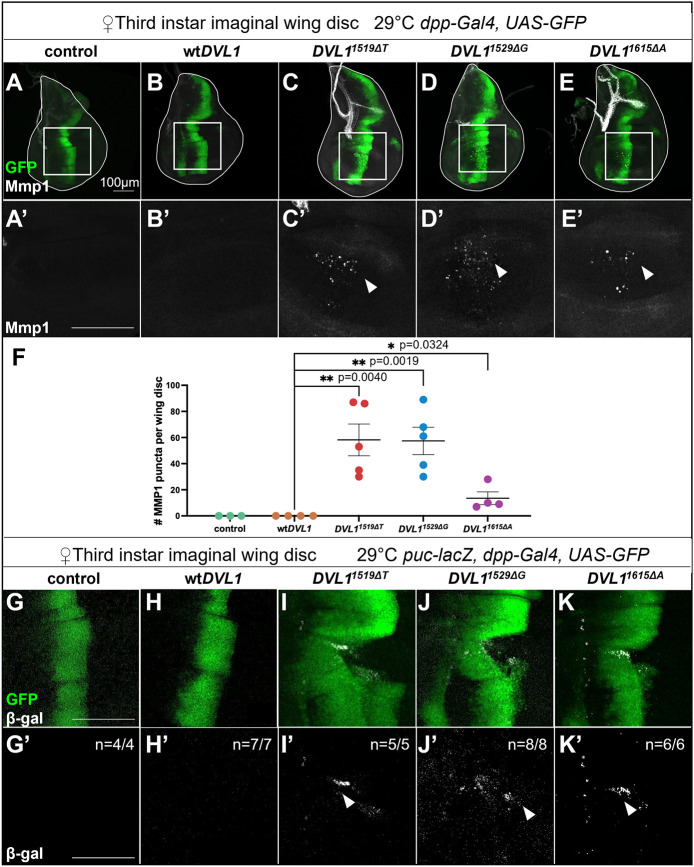
***DVL1* variants induce ectopic JNK signaling.** (A-E) *Z*-stack maximum-intensity projections of imaginal wing discs showing Mmp1 (white) and *dpp>GFP* expression domain (green) in control (A) and *DVL1* variant-expressing (B-E) female wing discs. (A′-E′) *Z*-stack maximum-intensity projections of the boxed regions in A-E showing Mmp1 protein (white) staining in control (A′) and *DVL1*-expressing female wing discs (B′-E′). Arrowheads in C′-E′ indicate Mmp1 puncta. (F) Number of Mmp1 puncta per wing disc. Crosses were performed at 29°C and three to five female wing discs were analyzed per genotype across one experiment. Data show the mean±s.d. Statistics were performed with one-way ANOVA and Dunnett’s post hoc test with all comparison of *DVL1* genotypes made to wt*DVL1*. *P<0.05; **P<0.01. (G-K′) *Z*-stack maximum-intensity projections of imaginal wing disc pouches showing β-galactosidase staining to detect *puc-lacZ* expression (white) and *UAS*-transgene expression domain (green) in control (G,G’) and *DVL1* variant-expressing (H-K′) female wing discs. Arrowheads in I′-K′ indicate areas of elevated *puc-lacZ* staining. *n-*values in the bottom row depict the number of wing discs that displayed the phenotype shown in the representative image. Crosses were performed at 29°C and four to eight female wing discs were analyzed per genotype across one biological replicate. Scale bars: 100 µm.

PCP defects in adult wing hairs can arise through both loss or gain of Wnt/PCP pathway activity (Axelrod et al., 1998; Krasnow and Adler, 1994; Vinson and Adler, 1987), thus making it impossible to conclude how signaling pathway activity is altered based on this phenotype. To investigate PCP-JNK signaling activity, we used two established JNK signaling reporters, the target genes *mmp1* and *puckered* (*puc*)*-lacZ*. Both assays indicated that the *DVL1* variants ectopically induced JNK signaling in the wing pouch (Fig. 7). There was no Mmp1 staining in controls or in wt*DVL1*-expressing wing discs (Fig. 7A-B′), whereas ectopic Mmp1 was detected in all the *DVL1* variant-expressing discs (Fig. 7C-F). Like the adult wing phenotypes, Mmp1 induction was more significant with *DVL1^1519ΔT^* and *DVL1^1529ΔG^* compared to that with *DVL1^1615ΔA^* under the same experimental conditions (Fig. 7F). These results were confirmed with the *puc-lacZ* transcriptional reporter (Fig. 7G-K′; Fig. S10A-E′). None of the control or wt*DVL1*-expressing discs exhibited active JNK signaling in the wing pouch (Fig. 7G-H′), whereas all the variants induced ectopic *puc-lacZ* expression in 100% of the wing pouches (Fig. 7I-K′). As the expression of JNK targets with the *dpp-Gal4* driver was mild, we increased expression of the variants with the stronger *apterous* (*ap*)*-Gal4* driver and observed a robust induction of *puc-lacZ* in *DVL1* variant-expressing discs but not in wt*DVL1*-expressing discs or controls (Fig. S10A-E′). Furthermore, we observed that the variant transgenes caused tissue distortions that made it difficult to distinguish between the dorsal and ventral compartments.

### *DVL1* variants disrupt *Drosophila* endogenous canonical Wnt/Wg signaling

**Fig. 8.**
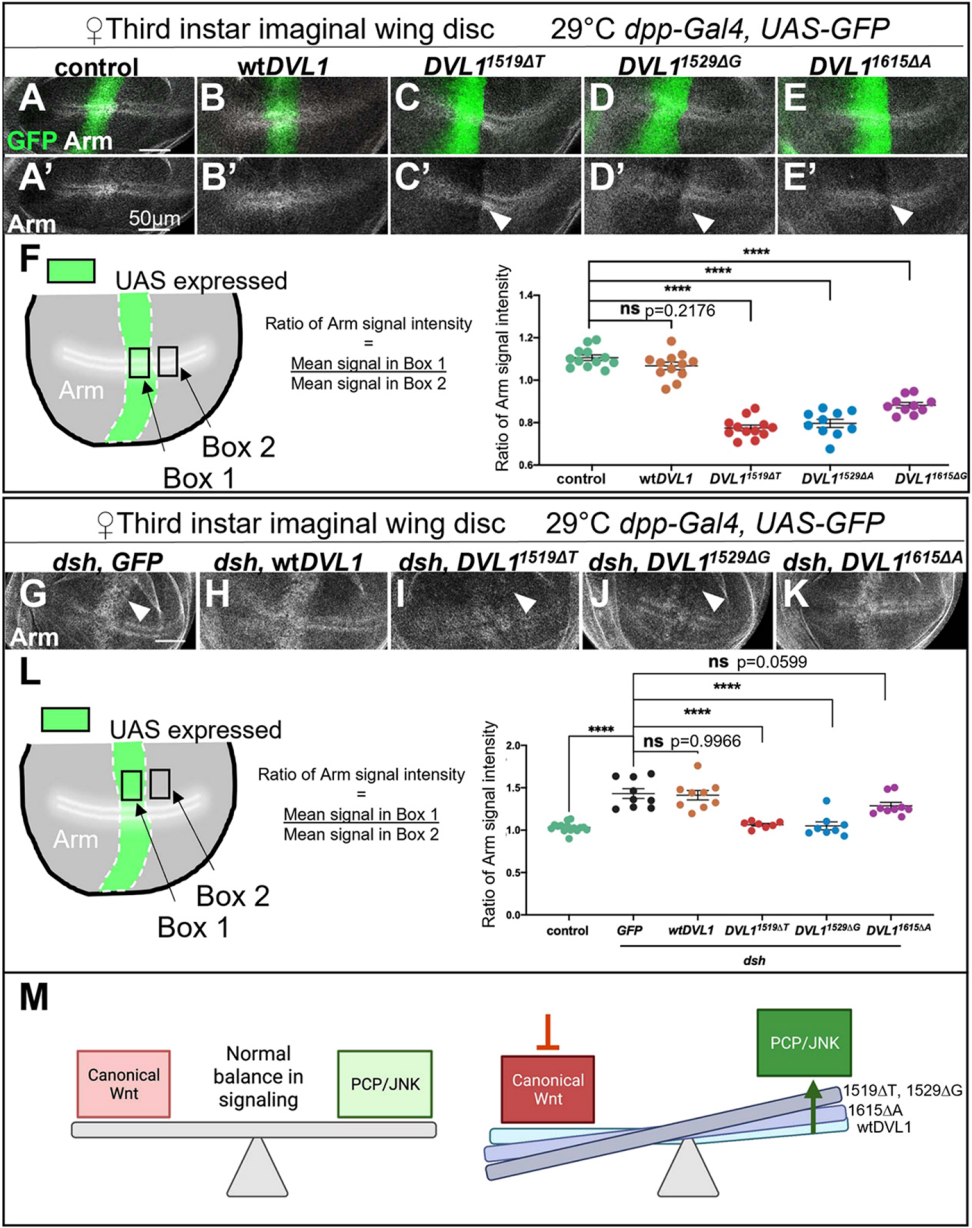
***DVL1* variants disrupt Armadillo stability.** (A-E′) Armadillo protein staining in wing imaginal discs with *dpp-Gal4>UAS-GFP* crossed to the indicated transgenes. *Z*-stack maximum-intensity projections of Arm protein (white) and *dpp>GFP* expression domain (green) in control (*dpp>GFP*) (A) and *DVL1*-expressing (B-E) female wing discs with single channel Arm staining shown in A′-E′. Significant decreases in Arm levels relative to those in control are indicated with arrowheads. (F) Schematic of the imaginal wing disc pouch with *dpp-Gal4* expression domain (green with dashed white outline) and the position of the stabilized Arm protein (white stripes). Boxes 1 and 2 show the regions where the relative ratio of Arm signal intensity was quantified (shown on the right) within and outside of the *dpp* expression domains. Crosses were performed at 29°C and 10-12 female wing discs were analyzed per genotype across two independent biological replicates. One-way ANOVA was carried out comparing all conditions to GFP controls, followed by Dunnett's post hoc test. (G-K) *Drosophila UAS-dsh* was co-expressed with the indicated human *DVL1* transgenes using *dpp>GFP*. *Z*-stack maximum-intensity projections with Arm (white) staining in *GFP*-expressing (G) and *DVL1*-expressing (H-K) female wing discs in a *dsh* overexpression background. Suppression of *dsh*-induced Arm stability is indicated with white arrowheads. (L) Quantification of the relative levels of Arm protein as described in F. Crosses were performed at 29°C and 7-15 female discs per genotype from two independent biological replicates. Scale bars: 50 µm. Error bars show mean±s.d. Statistics were performed with one-way ANOVA and Dunnett's post hoc test to *GFP* controls. (M) Schematic of the Wnt signaling imbalance induced by the DVL1 variants. ns, not significant; ****P<0.0001.

Although most of the known mutations associated with RS are in genes encoding components of the non-canonical Wnt/PCP pathway, both the canonical and non-canonical pathways are DVL1 dependent. As the HEK293 data suggested that variants were unable to activate canonical Wnt signaling and, moreover, had partial dominant-negative effects, we used the stability of Armadillo (Arm), the *Drosophila* ortholog of β-catenin, as a measure of canonical Wnt activity in the wing disc. Arm is stabilized after normal pathway activation and is classically seen as two stripes of elevated expression in the center of the pouch (Fig. 8A,A′). We observed a significant decrease in endogenous Arm protein levels when the *DVL1* variants, but not wt*DVL1*, were expressed (Fig. 8F), suggesting that canonical Wnt/Wg signaling is disrupted by the variants (Fig. 8B-E′, arrowheads).

We next tested whether the variants had any effect on the activity of elevated *Drosophila* Dsh. Consistent with published data, overexpression of *dsh* resulted in elevated ectopic Arm expression in the stripe of cells corresponding to the *dpp-Gal4* expression domain (Fig. 8G, arrowhead) (Yanagawa et al., 1997). Expression of wt*DVL1* had no effect on the ectopic Arm induced by *dsh* (Fig. 8H,L). However, both *DVL1^1519ΔT^* or *DVL1^1529ΔG^* could significantly suppress *dsh*-induced ectopic Arm, with Arm levels comparable to those of the control (Fig. 8I,J; L). This effect was not significant with that seen upon *DVL1^1615ΔA^* expression under these experimental conditions, likely due to lower protein levels (Fig. 8L; Fig. S8B,C). These data together show that all three variants can suppress endogenous Wg-induced Arm stabilization (Fig. 8A-E). Furthermore, two of the variants can interfere with the activity of elevated Dsh in the Wnt/β-catenin pathway *in vivo* (Fig. 8G-L) and suggests that the variants have dominant-negative activity.

## DISCUSSION

In this study, we investigated human mutations in *DVL1* that cause Robinow syndrome. These mutations are molecularly unusual in that they cause a frameshift, but instead of introducing a stop codon after a few base pairs, the sequence is translated and ∼141 amino acids are fused to the rest of the DVL1 protein (Table S6B; the DIX, PDZ and DEP domains are intact). In addition, there is striking conservation of the abnormal peptide sequence between individuals, even though the frameshift takes place in slightly different regions of exon 14 (Fig. 1A; Fig. S6B). The genetic data suggest that new patients are diagnosed with RS because *de novo* mutations are localized to the C-terminus rather than hitting the key functional domains of DVL1, DVL2 and DVL3. Furthermore, the mutant *DVL1* transcripts are not subject to nonsense-mediated decay and result in translated proteins (Fig. S8), and as others have shown in mouse cell lines (Bunn et al., 2015).

The novelty of our study is that we established two complementary model systems in which to probe gene function, the chicken embryo and *Drosophila*. We overexpressed human genes in addition to the endogenous copies found in the chicken and *Drosophila* genomes. Although the levels of expression of the exogenous genes were above physiological levels, the levels of the *Gallus DVL1* RNA were unchanged. This is important as we rule out a decrease in the levels of *Gallus DVL1* RNA mediating the phenotypes. There are likely normal levels of *Gallus* DVL1 protein expressed in the presence of the variants. In the fly, the human DVL1 proteins (wt and variants) did not affect intracellular distribution of fly Dsh nor the ability of Dsh to induce Arm expression in the presence of the wtDVL1. However, Dsh was blocked from stimulating Arm expression by the variant DVL1. Some normal functions of endogenous DVL1/Dsh took place in the presence of the transgenes, but there is also the possibility that the mutant protein competed with the normal protein in certain contexts. In these situations, the phenotypes would recapitulate those seen in an autosomal dominant, heterozygous RS mutation. Indeed, in the presence of viruses encoding *DVL1* variants, we saw novel skeletal phenotypes that included regional cartilage dysplasia, nuclear deformation and novel *Drosophila* phenotypes (e.g. folds in the wing, ectopic bristles and vein abnormalities).

### Effects of variant genes on canonical and non-canonical JNK-PCP Wnt signaling

Most reports describing human variants in RS genes do not provide mechanistic insights into the molecular effects of the gene mutations (Abu-Ghname et al., 2021; Conlon et al., 2021; Hu et al., 2022; Rai et al., 2021; Roifman et al., 2015; Shayota et al., 2020; White et al., 2015, 2018, 2016; Zhang et al., 2021). The majority of these studies have exclusively concluded that the JNK-PCP pathway is the main target of the mutations (Mazzeu and Brunner, 2020). There is only one study of RS by another group (DVL1) (Bunn et al., 2015) and our study on the WNT5A^C83S^ variant (Gignac et al., 2019). The WNT5A protein normally inhibits canonical Wnt signaling (Mikels et al., 2009) and we found in luciferase assays that the WNT5A^C83S^ variant could antagonize the intracellular canonical pathway when stimulated by LiCl (Gignac et al., 2019). If the WNT3A protein was added, the WNT5A^C83S^ variant was unable to antagonize the SuperTopFlash luciferase unless exogenous ROR2 receptor was added. The complex signaling defects caused by the WNT5A^C83S^ variant *in vivo* could vary depending on the tissue. For example, in the skeleton where there is abundant *ROR2* expression (Stricker et al., 2006), there could be lower canonical Wnt signaling.

The imbalance in Wnt signaling is clearer in the present study. We found that all *DVL1* variants lost most of the ability to stimulate canonical activity using luciferase assays. We went on to combine wt*DVL1* with mutant *DVL1* and revealed the dominant antagonism of the canonical Wnt reporter. Moreover, these results were confirmed and extended in *Drosophila* genetic studies in which the level of the Arm protein was decreased specifically when variant forms of human DVL1 were expressed. In a study by others, the *DVL1^1519ΔT^* variant was expressed in mouse myoblast C2C12 cells and found to activate canonical Wnt signaling when co-expressed with wt*DVL1* (Bunn et al., 2015). The unexpected activation could be due to their use of tenfold lower DNA in their transfections compared to ours (0.004 µg), which would mean that all readings were confined to the lowest range of detection. We found in *Drosophila* and HEK293 cells that three different *DVL1* variants decreased canonical Wnt signaling. The replication between variants, approaches and the *in vivo* studies in two animals suggests that at the cellular and molecular level, the RS DVL1 variant proteins exert dominant antagonism on the endogenous proteins. We recognize that further work is needed to determine the *in vivo* targets of the mutant *DVL1* genes as other pathways are likely to be impacted.

The wt*DVL1* activates the readouts of JNK-PCP signaling as shown by the loss of cell polarity in early chondrocytes, loss of the flattened shape and activation of the ATF2 luciferase reporter. Two of the variant forms also increased these JNK-PCP readouts to the same extent or greater than wt*DVL1*. We previously showed that similar effects were exhibited by the wt*WNT5A* and the *WNT5A^C83S^* variant on chondrocyte polarity, cell flattening and activation of the ATF2 reporter (Gignac et al., 2019). Therefore, we can rule out a loss of JNK-PCP signaling caused by the two of the most common mutations that cause RS. Moreover, *Drosophila* experiments showed that not only was JNK-PCP signaling maintained, but there was also significant activation of the PCP pathway using two different targets, Mmp1 and *puc*. Although most of the human genetic studies predicted disruption of the JNK-PCP pathway, we are the first to show a net gain in this pathway in *Drosophila* assays and selectively greater JNK-PCP activity by the *DVL1^1529ΔG^* variant in HEK293 cells and chicken limb chondrocytes. Taken together, our results suggest that there is an imbalance in the levels of signaling of the canonical and JNK-PCP pathways in patients with *DVL1* variants (Fig. 8M).

### Neomorphic phenotypes produced in chicken and fly may involve other signaling pathways

Our data supports the model that expression of *DVL1* variants causes acquisition of neomorphic phenotypes that are not seen after expressing wt*DVL1*. Chief among these readouts were the vein defects, ectopic bristles and wing blade creases in the *Drosophila* wing. The formation of the cross veins is dependent on BMP signaling (Montanari et al., 2022). Bristle specification requires numerous signaling pathways including the Notch pathway (Furman and Bukharina, 2011). The wing crease defects suggest that there are effects on epithelial adhesiveness. Thus, future studies will investigate whether the variants disrupt other pathways and processes in flies.

Consistent with a model of neomorphic functions, we noticed transcriptional changes in the chicken embryo chondrocytes. Some cells did not express *SOX9* at the appropriate time. The late differentiating cells would be out of sync with the rest of the morphogenetic program. This consistent finding of uneven GAG and SOX9 expression in the *DVL1* variant-infected limbs shows that the mutant proteins interfere with the initial cartilage condensations. The exact mechanism needs to be determined but could involve repression of *SOX9* by DVL1, which we and others showed can translocate to the nucleus (Fig. 5) (Habas and Dawid, 2005; Itoh et al., 2005; Sharma et al., 2021; Weitzman, 2005). Another novel phenotype we saw from the variants was that chondrocytes had deformed nuclei. In recent work, the mechanosensing properties of the cytoskeleton are connected to the nuclear membrane via the linker of the nucleoskeleton and cytoskeleton (LINC) complex and other protein complexes (Cantwell and Dey, 2022; Kalukula et al., 2022; Niethammer, 2021). As hypertrophic chondrocytes are not motile and not proliferating, the effects of nuclear membrane deformation remain to be determined. The correlation between the striking changes in nuclear shape and the translocation of DVL1 variants to the nucleus is also suggestive of an underlying relationship to the phenotype.

### Consistency of phenotypes between the RS variants across systems

Our experiments in the fly showed that all three RS variants of *DVL1* were expressed comparably at the mRNA level (Fig. S8A) and variably at the protein level, with the DVL1^1519ΔT^ and DVL1^1529ΔG^ variants more highly expressed than the DVL1^1615ΔA^ variant (Fig. S8B,C). Although *DVL1^1615ΔA^* consistently showed less severe phenotypes than those induced by the other variants, we were able to show that when expression of *DVL1^1615ΔA^* was increased, it could induce similar phenotypes as those induced by the other variants (Fig. 6H). Although we do not have an explanation for the differences in protein levels between the variants in this system, it is possible that as the *DVL1^1615ΔA^* frameshift is induced more downstream than in the *DVL1^1519ΔT^* and *DVL1^1529ΔG^* alleles, it could have retained regulatory sites that target it for degradation or inactivation, which are lost in the other more strongly expressed alleles. Despite this, the activity of all the variants consistently followed the same trends in our studies.

In the chicken embryo, there were also functional differences between the variants, particularly in the luciferase assays and the chondrocyte polarity assays, in which the *DVL1^1529ΔG^*-expressing virus had more activity. The early variability is due to lack of synchronization of viral infection of the mesoderm after injection of the particles.

### The role of the abnormal C-terminal peptide in mediating the effects of RS mutations

It is striking that only variants that lead to frameshifts in DVL1, DVL2 or DVL3 are found to cause RS (White et al., 2015, 2016; Zhang et al., 2021). Only one *de novo* missense variant in *DVL3* has been associated with RS, but this needs functional validation (Rai et al., 2021). The sequence of the novel DVL3 C-terminal peptide results in a frameshift mutation that gives rises to an abnormal C terminal peptide (Table S6A) (White et al., 2016). There is no homology to the abnormal DVL1 C-terminal peptide (Table S6B). If the peptide sequence itself was driving the RS phenotypes, then we predicted that it would translate to the same sequence in *DVL1* and *DVL3* frameshifted mutations. Thus, the sequence itself may not be the most important aspect of the mutant C-terminus.

We wondered whether the loss of the C-terminus causes abnormalities in signaling. One study removed the C-terminus by introducing a stop codon after nucleotide 1519 and found that the shorter version of *DVL1* was able to activate SuperTopFlash but to a lesser degree than wt*DVL1* (Bunn et al., 2015). Therefore, it appears that loss of the C-terminus may partially reduce levels of canonical signaling, but this needs further work to confirm the results in HEK293 cells.

Rather than loss of specific functions of the C-terminus, the gain of the abnormal peptide is likely to have a major impact on DVL1 function. Other possible effects of the frameshift mutations are abnormal protein folding (Hu et al., 2022; Lee et al., 2015) or trafficking (shown in the immunocytochemistry results). We did not observe that the variants themselves correlate to severity of phenotypes as we only detected some minor differences between variants in the chicken embryo at stage 34, *Drosophila* and HEK293 luciferase assays, but, overall, the general results were similar. The variation in clinical phenotypes among patients with the same mutation is due to the genetic background of the individual (Abu-Ghname et al., 2021; Conlon et al., 2021; Schwartz et al., 2021). Although some efforts have been made to carry out genotype-phenotype correlations in patients diagnosed with RS (Zhang et al., 2022), there are still too few individuals studied in enough detail to make solid conclusions.

In conclusion, our studies provide molecular insight into a rare disease and the function of DVL1. The study of RS has highlighted important roles of the *DVL1* gene in skeletogenesis and PCP-JNK activity. More broadly, our study highlights how important it is to use multi-pronged approaches when embarking on functional genomics studies on human gene variants.

## MATERIALS AND METHODS

### Cloning of human *DVL1* wild-type and variant genes

The open reading frame encoding human *DVL1* (Origene RC217691) was originally in the pCMV6 vector (Origene-provided clone in this vector) with the full coding sequence and two tags, myc and Flag. We moved *DVL1* into pDONR221 (Invitrogen) using Gateway cloning. All site-directed mutagenesis was performed in those clones and then recombined into the pENTRY destination vector (Invitrogen) with LRclonase2 (Life Technologies). Three mutations that cause autosomal dominant RS type II were generated (OMIM: 616331) using site-directed mutagenesis with restriction-free cloning (Bond and Naus, 2012): the 1519ΔT, 1529ΔG and 1615ΔA mutations of the *DVL1* gene resulted in a frameshift mutation. A stop codon was added to the 3′ end of the coding sequences for wt*DVL1* as there were C-terminal tags that were added by the company (Origene). The mutant constructs resulted in a natural stop codon owing to the frameshift. Gateway cloning (Invitrogen) was used to move the mutant or wild-type sequences from pENTRY into compatible destination vectors (Loftus et al., 2001). The Gateway-compatible RCASBPY retrovirus [replication-competent avian sarcoma-leukosis virus (ASLV) long terminal repeat (LTR) with splice acceptor (RCAS)] (Loftus et al., 2001) was used to deliver human genes to the avian embryo. The RCASBPY retrovirus was a gift from Stacie Loftus, National Institutes of Health. The following RCASBPY constructs were created for the *in vivo* chicken experiments: wt*DVL1*, *DVL1^1519ΔT^*, *DVL1^1529ΔG^* and *DVL1^1615ΔA^*. The same gene inserts were moved into pcDNA3.2/V5-DEST (Invitrogen) for *in vitro* plasmid transfections used in luciferase assays. The *GFP*-expressing virus was generously provided by another investigator (Stephen J. Gaunt, University of Cambridge) and has been used in other studies from our lab (Higashihori et al., 2010; Nimmagadda et al., 2015).

Flag-tagged versions of DVL1 were created to track the protein in *Drosophila* studies as well as in immunocytochemistry studies on HEK293 cells. A Kozak sequence followed by an N-terminal Flag tag was cloned upstream of the coding sequence for *DVL1*. The plasmids used in immunocytochemistry were cloned into pcDNA3.2 and included the sequences for Flag-wtDVL1, Flag-DVL1^1519ΔT^, Flag-DVL1^1529ΔG^ and Flag-DVL1^1615ΔA^. The same Flag-tagged forms of DVL1 were cloned into the *Drosophila* pUASg_attB_Gateway vector (a gift from Johannes Bischof, Institute of Molecular Life Sciences, Zurich, Switzerland).

### Avian retrovirus propagation

Retroviruses were propagated by using the DF1 chicken cell line (American Type Culture Collection, CRL-12203) as described previously (Logan and Francis-West, 2008) and collected after 6-8 weeks of culture. Cells were grown in Dulbecco's modified Eagle medium (Invitrogen), fetal calf serum (10%; Sigma-Aldrich), 1× penicillin/streptomycin and 1× glutamine. Prior to transfection of the proviral plasmids, DF1 cells were checked for contamination by other RCAS virus using qRT-PCR for *GAG* RNA. Viruses encoding the wild-type and mutant *DVL1* genes did not affect the growth of the parent cell line, DF1 fibroblasts (similar passaging times). We confirmed that *DVL1* genes encoded in the viruses were expressed in the injected limbs by using a human-specific *DVL1* primer set within exon 7 (Fwd: 5′-CAGCATAACCGACTCCACC-3′; Rev: 5′-TGATGCCCAGAAAGTGATGTC-3′) (Fig. S1A). Only the limbs injected with human *DVL1-*expressing viruses contained the *DVL1* amplicon. There were three biological replicates collected at 72 h (stage 28) consisting of three limb buds pooled in each sample. SYBR Green (Bio-Rad)-based qRT-PCR was carried out using an Applied Biosystems StepOnePlus instrument (95°C for 5 s, 60°C for 20 s, 40 repeats). Levels of expression were normalized to 18S RNA (Applied Biosystems, 4318839), as we have used in previous chicken studies (Gignac et al., 2019; Hosseini-Farahabadi et al., 2017; Nimmagadda et al., 2015). The ΔΔCt method was used to calculate relative fold-change expression between wt*DVL1-* and the variant-infected limbs (Schmittgen and Livak, 2008). One-way ANOVA in reference to wt*DVL1* followed by Dunnett's multiple comparison test was used to determine significant differences in fold-change expression between variants and wt*DVL1*. To assess the effect of the human genes on endogenous *Gallus DVL1*, primers specific to *Gallus DVL1* were used to amplify the sequence (Fwd: 5′-CTCCCATTGAGAGGACAGGT-3′; Rev: 5′-TGTTTCGTTGTCCAGTCCAT-3′). One-way ANOVA was carried out without a defined control group followed by Tukey's multiple comparison test.

### Chicken embryo experiments

White leghorn eggs (*Gallus gallus*) received from the University of Alberta were incubated to the appropriate embryonic stages, according to the Hamburger and Hamilton staging guide (Hamburger and Hamilton, 1951, 1992). Neutral Red stain (0.33%; Thermo Fisher Scientific, N129) was used to enhance visualization of the embryos while staging. Work on prehatching chicken embryos is exempt from ethical approval by the University of British Columbia Animal Care Committee and the Canadian Council on Animal Care.

#### Retrovirus injection into the chicken limb field and analysis of phenotypes

The limb field of stage 15-16 chicken embryos (25-28 somites) was injected with concentrated RCAS retrovirus viral particles as described previously (Gignac et al., 2019). Embryos were grown until stage 38, fixed in 100% ethanol and processed for bone and cartilage staining with Alcian Blue (Sigma-Aldrich) and Alizarin Red (Sigma-Aldrich) as described previously (Lee et al., 2001).

#### Histology and immunofluorescence staining

A different set of chicken embryos was injected with viruses at stage 15 and fixed at stages 28, 29 or 34 with 4% paraformaldehyde for microscopic studies as described previously (Gignac et al., 2019). Stage 34 embryos were decalcified in 12% EDTA (Thermo Fisher Scientific, S312-212) at 4°C on a shaker for 4 days prior to processing into paraffin. Selected sections were stained with Alcian Blue and Picrosirius Red (Sigma-Aldrich) to detect differentiating cartilage and bone as described previously (Danescu et al., 2015). Immunofluorescence analysis was carried out at various stages of development using protocols described in Table S5. Fluorescence images were acquired with a 20× objective on a slide scanner (3DHISTECH, Budapest, Hungary).

#### Histomorphometry, nuclear shape, chondrocyte polarity and cell shape

Stage 34 limb sections were stained and scanned in bright-field or fluorescence mode to see the Hoechst 33258 (Sigma-Aldrich)-stained nuclei. The length in the proximo-distal axis and anterior-posterior axis (measured in the center of the diaphysis, perpendicular to the long axis of the bone) for each bone was measured for each embryo or biological replicate. The nuclear shape was assessed in the diaphysis across a full field of view at 63× (Histech slide scanner) or 4.8×10^4^ µm^2^. ImageJ software was used to set the image to 8 bit, set the contrast, set the threshold, analyze particles (settings: 200-∞, outlines) and then measure. Circularity was used as the readout. In the same field of view, nuclei were manually scored as being normal (oval) or abnormal (triangular, polygonal, star-shaped or rectangular). The percentage of cells in each category was determined in three biological replicates. One-way ANOVA and post hoc test was used to determine significance between comparisons. Embedded in the one-way ANOVA analysis is Bonferroni multiple testing correction.

Stage 29 embryos were used to measure chondrocyte polarity and chondrocyte shape. Golgi angles between 0° and 90° (Golgi-nucleus angle relative to the long axis of the cartilage in the diaphysis region of the forelimb zeugopod) were analyzed in an area of 400 µm^2^ in the middle of the ulna containing on average 63 cells. Angle orientation was plotted in ImageJ. Cell shape was measured by circumscribing the Prickle-stained regions using the polygon tool in ImageJ. Aspect ratio (height/width) values close to 1 indicate that the cell has a circular shape.

#### BrdU and TUNEL analysis in chicken embryos

Apoptosis was analyzed using terminal deoxynucleotidyl transferase dUTP nick-end labeling (TUNEL) staining at stage 29 using the ApopTag Apoptosis Kit (Chemicon, S7101). For cell proliferation studies, 96 h post-virus-injection (stage 29) embryos were labeled with 1 µl of 10 mM BrdU (Sigma-Aldrich, B5002) injected into the heart 2 h prior to euthanasia. Staining was performed as detailed in Table S5. The proportion of BrdU-labeled cells in the ulna was manually determined using ImageJ. The proportion of BrdU-positive cells to total cells was determined in the entire ulna of the developing forelimb (*n*=3 embryos per virus genotype).

### *In vitro* studies on HEK293 cells

#### Luciferase assays to measure Wnt signaling activity

Luciferase assays were performed on HEK293 cells (American Type Culture Collection). Transient transfections were performed at 30-40% confluence (0.17-0.18×10^6^ cells/ml). Cells were transfected with plasmids using Lipofectamine 3000 (Invitrogen, L3000-008) 24 h after plating in 24-well plates (Invitrogen, L3000-008; Nunc, 142475). Plasmids containing the wild-type or variant *DVL1* genes along with firefly reporter plasmids for SuperTopFlash (M50 Super 8× TOPFlash was deposited by Randall Moon; Addgene plasmid #12456) and ATF2 (Ohkawara and Niehrs, 2011) were used as reported (Geetha-Loganathan et al., 2014; Gignac et al., 2019; Ohkawara and Niehrs, 2011). Luciferase assays were performed after 48 h of culture using the dual-luciferase reporter assay system (Promega, E1910). A Tecan luminometer (Spark multimode Tecan plate reader) was used to read luminescence activity at 1 s reading with an OD1 filter. At least three technical and three biological replicates were carried out for each transfection mixture and the experiment was repeated on two different days.

#### Immunocytochemistry on HEK293 cells expressing Flag-tagged DVL1 variants

HEK293 cells were cultured on coated coverslips (poly-L-lysine; Sigma-Aldrich, RNBC8085). Cultures were grown to 40% confluency and transfected using Lipofectamine 3000 (2.5 µg DNA; Invitrogen, L3000-008). Anti-Flag staining was carried out 48 h after transfection (Table S5). Three coverslips or biological replicates for each genotype were imaged using a slide scanner (3DHISTECH, Budapest, Hungary). Biological replicates are the average of cell counts made in five to six areas of 400 µm^2^ per coverslip. Approximately 50 cells are in each sampled region.

### *Drosophila* experiments

*Drosophila melanogaster* flies were raised on standard media; stocks were kept at room temperature and crosses were reared at 25°C or 29°C as indicated. Four *Gal4* fly lines were used to induce transgene expression: *dpp-Gal4, UAS-GFP*/*TM6B* (Swarup et al., 2015), *Dll-lacZ/Cyo; Hh-Gal4/TM6B* (Hall et al., 2017), *tj-Gal4/tj-Gal4* (a gift from Dr Nicholas Harden, Simon Fraser University; Vlachos et al., 2015), and *apterous* (*ap*)*-Gal4* (Bloomington *Drosophila* Stock Center, 3041). Additional stocks used were: *puc-LacZ* (Martin-Blanco et al., 1998) and *UAS-dsh* (Bloomington *Drosophila* Stock Center, 9453). As controls, the *Gal4* drivers were crossed with *w^1118^* or *UAS-GFP* flies. To generate transgenic *UAS* stock lines, patient variant Flag-tagged *DVL1* constructs were sent for integration into the attP40 locus on the second chromosome for generation of stably integrated fly strains (BestGene, CA, USA).

#### Immunofluorescence staining, microscopy and image processing on *Drosophila* tissues

Imaginal wing discs from female third instar larva were dissected in PBS and fixed in 4% paraformaldehyde at room temperature for 15 min. Samples were washed twice for 10 min with PBS containing 0.1% Triton X-100 (PBST). Following a 1-h block with 2-5% bovine serum albumin diluted in PBST at room temperature, samples were incubated overnight with primary antibodies at 4°C. The following primary antibodies were used: mouse anti-Flag (1:500, Sigma-Aldrich, M2; used for all experiments except co-staining with Dsh in ovarian follicle cells; Fig. S9F-J′), rabbit anti-Flag (1:500, Sigma-Aldrich, SIG1-25; used only in Fig. S9F-J′) and mouse anti-Myc (clone 4A6, 05-724, Millipore). All other clones were purchased from Developmental Studies Hybridoma Bank (DSHB; mouse anti-Armadillo, 1:50, clone N27A1; mouse anti-β-galactosidase, 1:50, clone 40-1a; mouse anti-Mmp1, 1:100 3A6B4, 3B8D12 and 5H7B11). Samples were washed twice for 10 min with PBST and incubated with Cy3- and/or Alexa Fluor 647-conjugated secondary antibodies (1:500, Jackson ImmunoResearch Laboratories; anti-mouse Cy3, 715-165-151; anti-mouse Alexa Fluor 647, 715-605-151) and DAPI (1:1000, Invitrogen, D1306) for 2 h at room temperature. After two 10-min washes, samples were mounted in 70% glycerol in PBS and imaged using a Nikon Air laser-scanning confocal microscope or a Zeiss LSM880 with Airyscan confocal microscope. Images were processed with FIJI software (Schindelin et al., 2012) and are presented as *z*-stack maximum-intensity projections unless otherwise stated.

#### Quantification of Arm protein levels

Imaginal wing discs were subjected to the immunofluorescence protocol described above. Following imaging, maximum-projection images were processed with FIJI software (Schindelin et al., 2012). Using a box of identical dimensions, the mean Arm signal intensity was quantified inside and outside of the transgene expression domain either on the most stabilized stripes of Arm protein or slightly above them as indicated. The ratio of Arm signal inside to that outside of the transgene expression domain was used to determine loss or gain or Arm levels within the transgene expression domain.

#### Adult wing mounting and imaging

Adult female fly wings were dissected in 95% ethanol and mounted in Aquatex (EMD Chemicals). Slides were baked overnight at 65°C. The wings were imaged with a Zeiss Axioplan-2 microscope and processed with ImageJ software. The areas of transgene expression domain between longitudinal veins L3 and L4 were quantified using Adobe Photoshop CS3.

#### qRT-PCR on imaginal discs

A total of ten salivary glands were dissected from five third instar larvae and pooled together to make one biological replicate. Three or four independent biological replicates were collected. RNA was isolated using RNeasy Plus Mini Kit (QIAGEN, 74134). cDNA was synthesized with OneScript Plus cDNA Synthesis Kit (Applied Biological Materials, G236). qRT-PCR were performed in triplicate using SensiFast SYBR Lo-Rox Kit (Bioline, 940004, 940020 or 940050) on a QuantStudio3 PCR machine (Thermo Fisher Scientific). We used the same human *DVL1* primers as were described in the chick methods. *rp49* (Fwd: 5′-AGCATACAGGCCCAAGATCG-3′; Rev: 5′-TGTTGTCGATACCCTTGGGC-3′) and *gapdh1* (Fwd: 5′-TAAATTCGACTCGACTCACGGT-3′; Rev: 5′-CTCCACCACATACTCGGCTC-3′) were used as housekeeping controls.

#### Western blotting for *Drosophila* salivary glands

*Drosophila* salivary glands were used for western blotting owing to the increased yield of protein compared to that from imaginal discs. Salivary glands were dissected and lysed by vortexing the samples for 30 s in 1× SDS sample buffer, which were then boiled for 5-10 min. The equivalent of two salivary glands of protein was then resolved on 10-12% SDS-PAGE gels before being transferred to nitrocellulose membranes. Following the transfer, membranes were blocked in 5% skimmed milk in TBS containing 0.1% Tween-20 and then incubated with primary and secondary antibodies. The following primary antibodies were used: mouse anti-Flag (1:1000, Sigma-Aldrich, M2), mouse anti-β-tubulin (1:1000, Abcam, G098) and mouse anti-actin (1:1000, Abcam, ab3280). Anti-mouse HRP (1:5000, Jackson ImmunoResearch Laboratories, 115-035-174) secondary antibody was used. Membranes were visualized using Clarity Western enhanced chemiluminescence (ECL) Substrate (Bio-Rad, 170-5061), imaged on a GE AI 600 Imager, and band density/protein levels on three independent blots were determined with FIJI software (Schindelin et al., 2012).

### Statistical analysis

All statistical analyses for limb length, cell shape, nuclear localization of Flag-DVL1, orientation of chondrocytes, BrdU, qRT-PCR (chicken and fly) and western blot band density were done using one-way ANOVA followed by Tukey's post-hoc test for multiple comparisons with Bonferroni correction. For luciferase assays, data were normalized to control pcDNA3.2 parent plasmid values and were analyzed by one-way ANOVA followed by Tukey's post hoc test with Dunnett's correction for multiple testing. For Mmp1 puncta and Arm expression, we used one-way ANOVA with Dunnett's post hoc test. We compared the Mmp1 puncta data to wtDVL1 in Fig. 7F and the Arm expression levels to GFP controls in Fig. 8F,L. All statistical analyses were performed using GraphPad Prism 9.3.1 to 9.5 (GraphPad Software, San Diego, CA, USA). Analyzed data with *P*<0.05 was considered statistically significant. Significance depicted as: ns, not significant; *P<0.05; **P<0.01; ***P<0.001; ****P<0.0001.

## Supplementary Material

10.1242/dmm.049844_sup1 Supplementary information
