# Supplementary material for: Mechanistic studies in Drosophila and chicken give new insights into functions of DVL1 in dominant Robinow syndrome
Source: Dis Model Mech. 2023 Apr 13;16(4):dmm049844. doi: 10.1242/dmm.049844 (PMC10120075; doi:10.1242/dmm.049844)
Supplement: Supplementary information [file dmm-16-049844-s1.pdf]

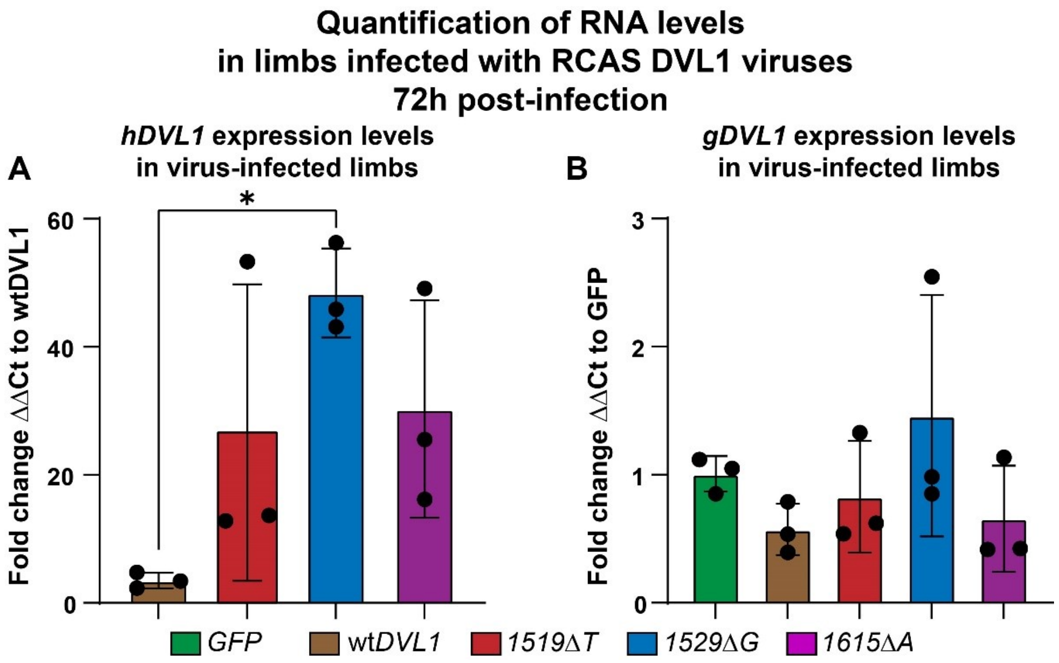

Fig. S1. Expression levels of exogenous human *DVL1* genes or endogenous gallus *DVL1* in vivo in the chicken limb bud

Limb buds were injected at stage 15 or E2.5 as for the retroviral infection experiments and RNA extracted 72h post-infection or stage 28. **A)** The primer set amplified a region of the human coding sequence between bp714 and 793, which is5’ to the frameshift mutations. Levels of expression were compared to wt*DVL1* using ΔΔCt. The *1529ΔG* variant had significantly higher expression than wt*DVL1* (1-way ANOVA relative to wt*DVL1*).

B) Measurement of gallus *DVL1* with gallus-specific primers shows no significant difference in any of the multiple comparisons (1-way, ANOVA, all groups compared to each other).

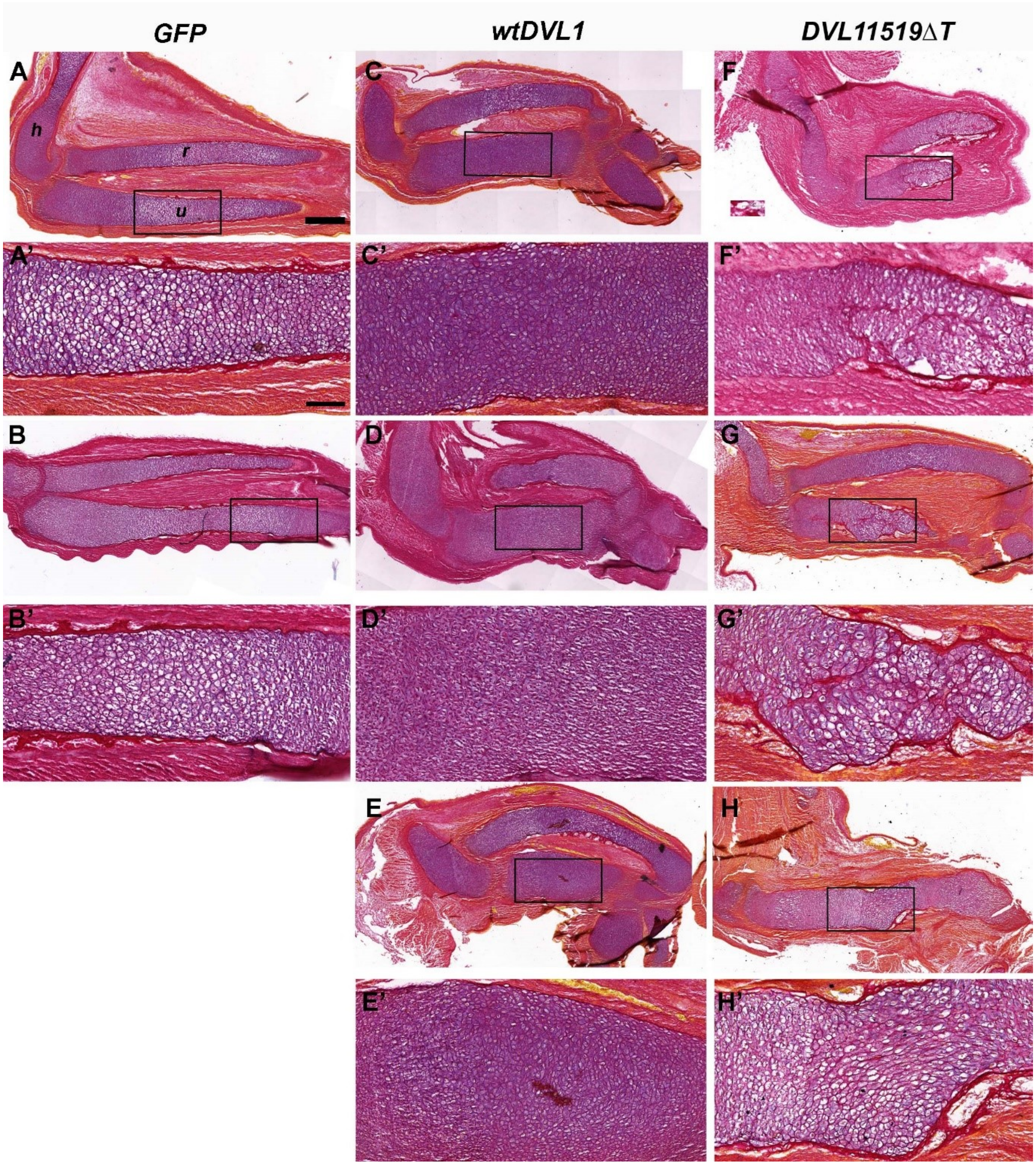

Fig. S2. Additional examples of specimens infected with RCAS viruses at stage 15 and collected at stage 34.

A-B') GFP control virus does not affect normal skeletogenesis. At this stage the chondrocytes in the diaphysis are enlarging thus decreasing cell density (Box in A, A'). In the diaphysis the density is increased since chondrocyte hypertrophy happens later. C-E') Three different embryos infected with *wtDVL1.* Chondrocyte hypertrophy is not present in the centre (Boxes) of the shortened and thickened cartilage rods. The cartilage elements have smooth borders. F-H') The *1519ΔT* variant caused regional disorganization of the cartilage including irregular borders of the cartilage rod (Boxes in F,G,H). Chondrocytes have varying sizes indicating that hypertrophy is dysregulated. There is a loss of organization. Scale bars = 400 µm for all low power views and 100 µm for high power views.

Disease Models & Mechanisms • Supplementary information

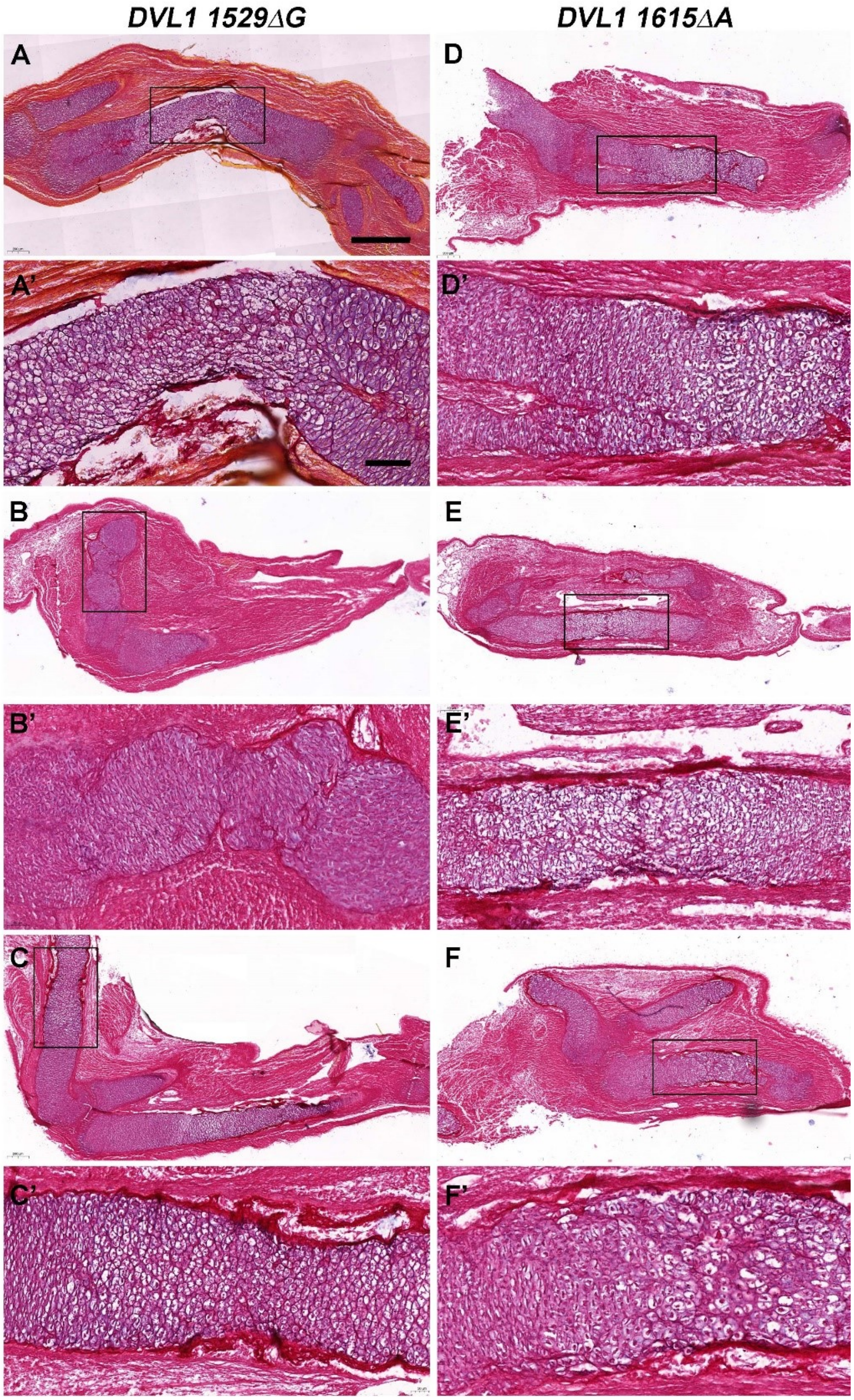

Fig. S3. Additional examples of specimens infected with RCAS viruses at stage 15 and collected at stage 34.

A-C') examples of limbs infected with the *1529ΔG* variant. Disorganization of cartilage occurs in bones where virus is expressed (see boxes). D-G') The 1*615ΔA*variant causes similar phenotypes in the cartilage to the other variants. Scale bars = 500 µm for low power views and 100 µm for high power views.

Disease Models & Mechanisms • Supplementary information

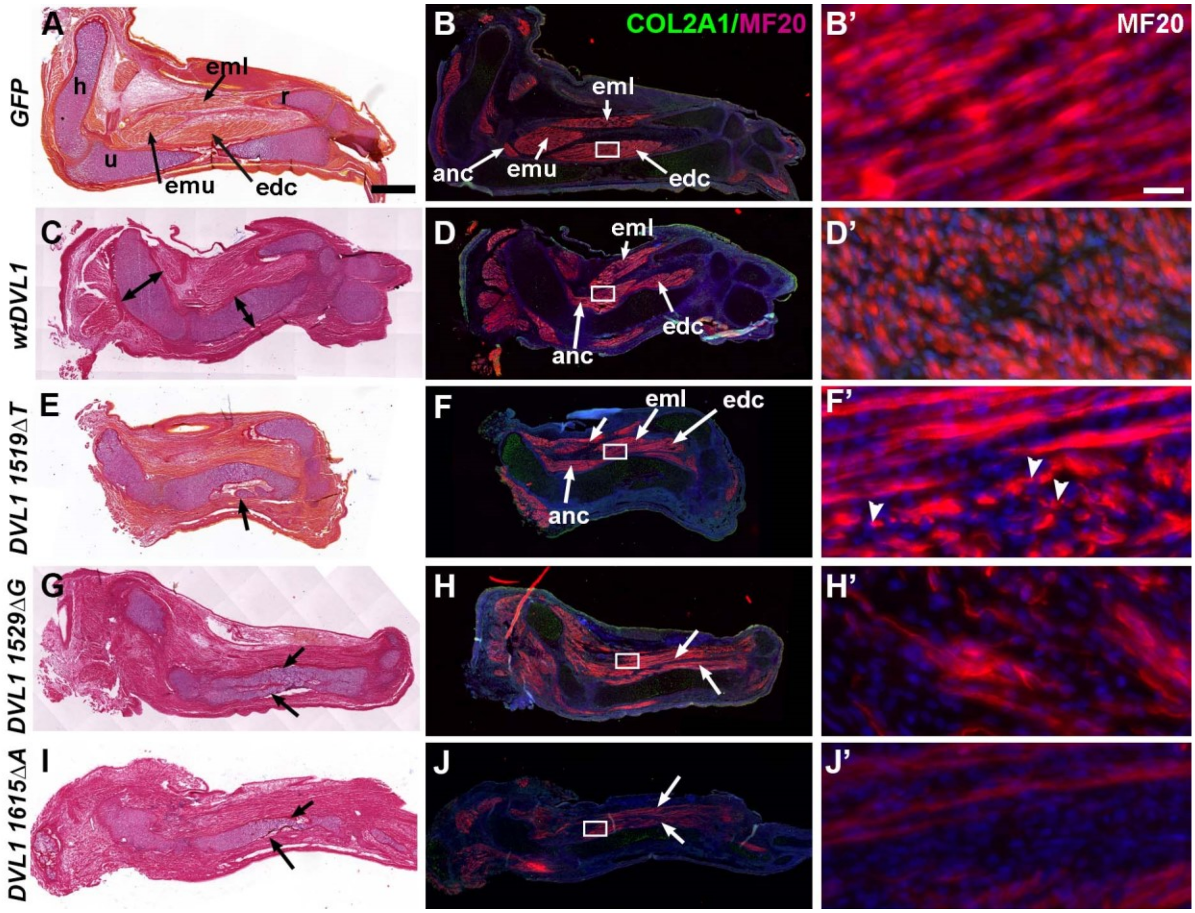

Fig. S4. Muscle abnormalities caused by DVL1 variants. Near-adjacent saggital sections of limbs at stage 34 stained with H&E or antibodies to type II collagen and muscle myosin (MF20). In GFP controls, well-delineated muscles are present (A-B'). Normal banding is present in muscle fibres (B'). In limbs infected with wt*DVL1* muscles are present but shorter than normal (C-D'). In the *1519ΔT* variant infected limbs it is more difficult to distinguish muscle anatomy (E-F'). The muscle fibres appeared to be oriented perpendicular to the long axis of the muscle (arrowheads, F'). In the *1529ΔG* (G-H') and *1615ΔA* (I-J'), muscle sheaths are not well formed and distinct muscles are not visible. Muscle fibres are not well organized (H', J'). Scale bar = 500 microns for low power views in columns 1 and 2. In column 3, scale bar = 20 µm. Key: anc – anconeus, edc – extensor digitorum communis, eml – extensor medius longus, emu – extensor metacarpi ulnaris, h – humerus, r – radius, u – ulna.

Disease Models & Mechanisms • Supplementary information

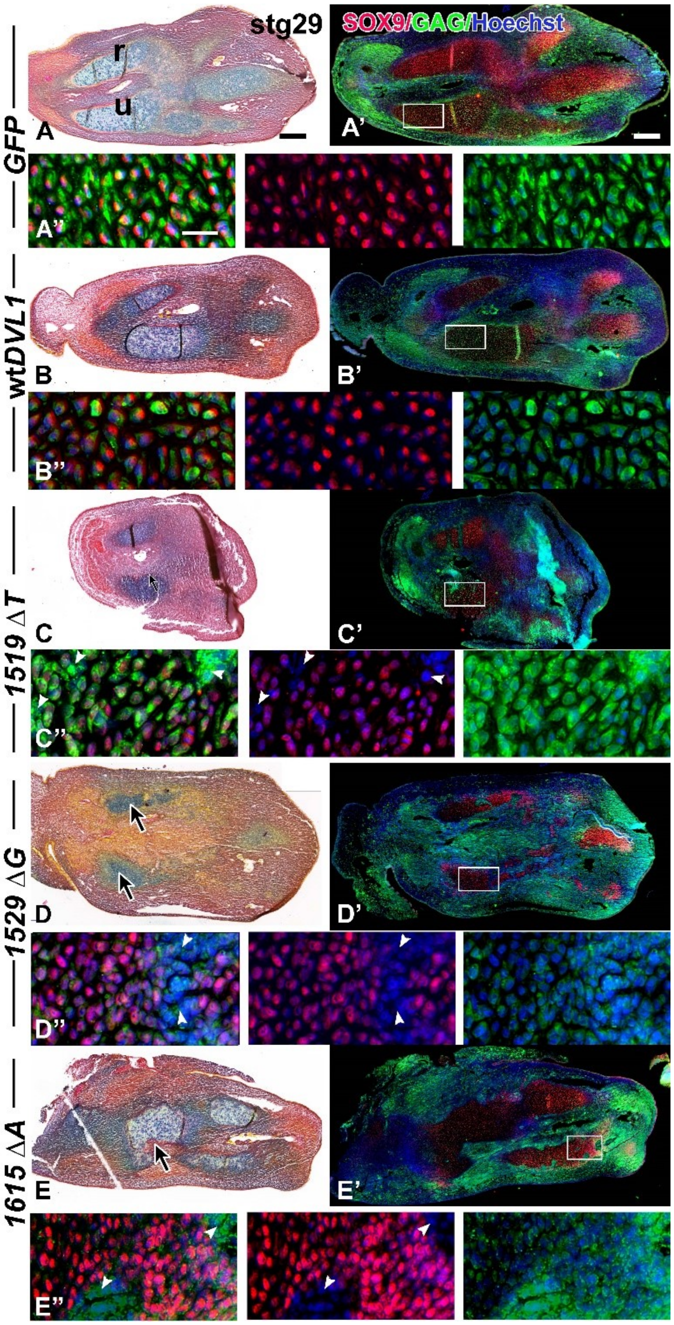

Fig. S5. Inhibition of chondrogenesis by DVL1 variants contributes to dysmorphology

(A) The chondrocytes are fully specified in stage 29 limbs and structure of the main skeletal elements is established. **(A',A'')** SOX9 staining confirms that chondrocytes are specified. There is overlap between GAG and nuclear staining for SOX9. (**B)** normal initiation of skeletal patterning in limbs infected with wt*DVL1*. (**B'-B'')** Full overlap between virus and differentiated chondrocytes expressing SOX9. (**C)** Cartilage elements are shorter in the $DVL1^{1519\Delta T}$ variant-infected limb. **(C'-C'')** Some areas of the cartilage have GAG staining but cells are undifferentiated as shown by the absence of SOX9 staining (white arrowheads). (**D, E)** The $DVL1^{1529\Delta G}$ and $DVL1^{1615\Delta A}$ variants have very dysmorphic cartilage elements. (**D'-D'', E'-E'')** Pockets of undifferentiated cells are within the cartilage elements (white arrowheads). Key: r – radius, u – ulna. Scale bar for low magnification images = 200 µm and 20 µm for high power images.

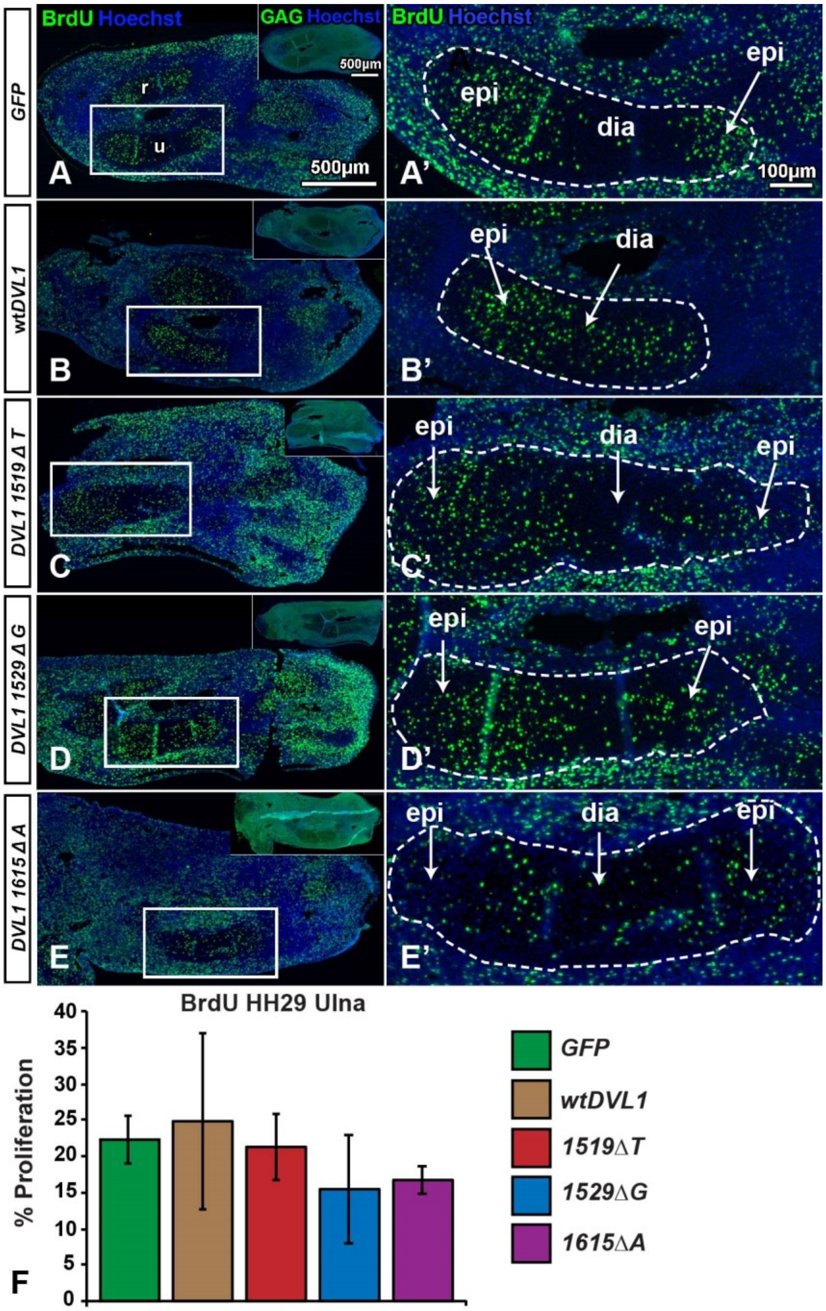

Fig. S6. Proliferation quantification following infection with wild-type *DVL1* and *DVL1* variants injected forelimbs. Embryos were injected with viruses into the limb bud at stage 15 and fixed at stage 29, 4 days post-infection. BrdU was injected 2h prior to euthanasia. A-E) Adjacent sections are stained with anti-BrdU labeling (green) or GAG to show levels of viral expression (insets). A'-E') show increased magnification the developing ulna (dashed line). (A,A') BrdU label was noticeably lower in the differentiating diaphysis in GFP controls. B,B') wtDVL1 had even BrdU labeling, consistent with delayed hypertrophy in the diaphysis. (C-E') Most of the mutant limbs had such irregular morphology that it was difficult to identify the borders of the epiphyses and diaphysis. F) BrdU positive cells in the entire ulna (inside dashed line) were quantified. No overall difference in cell proliferation was detected. One-way ANOVA, Tukey's post hoc test, n=3 for each virus. Scale bar=500 µm for A-E and 100 µm for A'-E'. Key: dia – diaphysis, epi – epiphysis, r – radius, u – ulna.

Disease Models & Mechanisms • Supplementary information

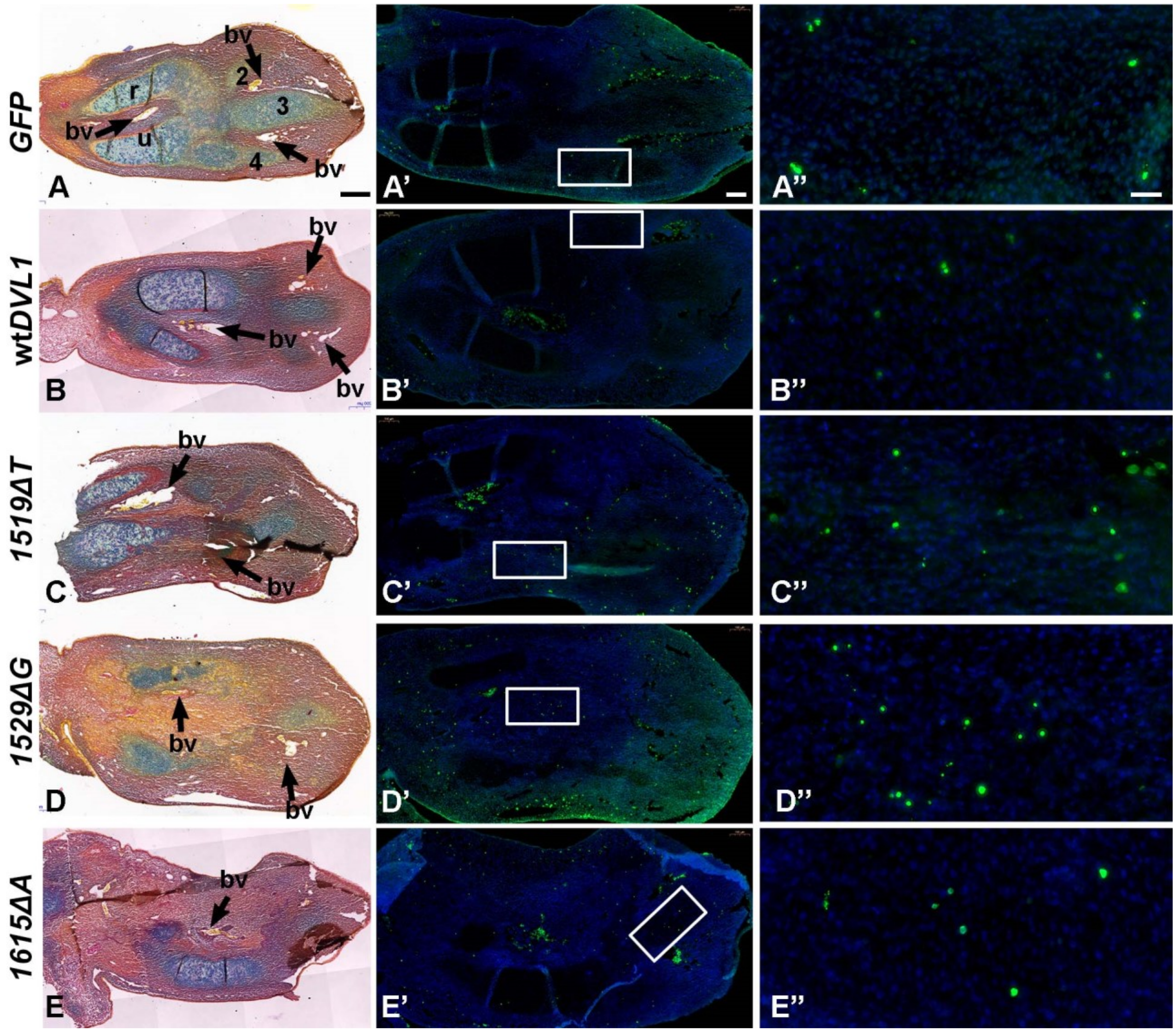

Fig. S7. TUNEL staining of limbs infected with RCAS viruses.

All embryos were injected at stage 15 and fixed at stage 29 (4 days later or E6.5). The anterior edge is uppermost and distal tip of the limb faces right. The first column are near-adjacent sections stained with Alcian blue and Picrosirius red. Gaps in the tissue are where large blood vessels are located. A'-E'') TUNEL stained sections. The right column shows inset in white box. Note, blood cells autofluoresce green in the green channel. There are very few TUNEL positive cells in the areas with cartilage. Normal apoptosis is seen along the anterior and posterior edges of the limb. Key: bv – blood vessel, r – radius, u – ulna, 2, 3,4 – digit numbers. Scale bars – 200 microns A-E, 500 microns A'-E and 20 microns A''-E''.

Disease Models & Mechanisms • Supplementary information

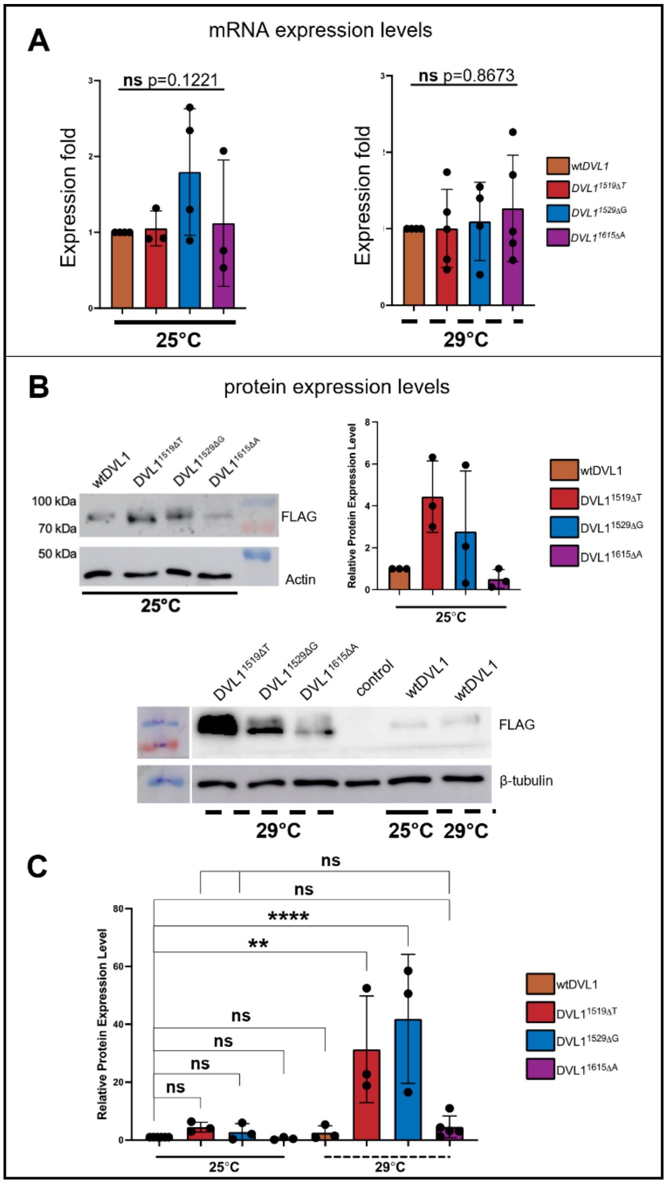

Fig. S8. UAS-DVL1 transgenes expression.

(A) mRNA expression of DVL1 transgenes expressed with dpp-Gal4 (dpp>DVL11519ΔT, dpp>DVL11529ΔG, and dpp>DVL11615ΔA) relative to control (dpp>wtDVL1). Crosses were performed at 25°C and 29°C and expression was normalized to two housekeeping genes, rp49 and gapdh1. All experiments were performed in triplicate. n=3 or 4 independent biological replicates per genotype, with samples comprising 10 salivary glands per genotype. Error bars = mean ± s.d.. (B) Representative 25°C and 29°C Western blots showing DVL1 protein levels from salivary gland extracts. dpp-Gal4>DVL1 crosses were performed at 25°C or 29°C as indicated. Salivary glands were harvested from W1118 flies as a negative control. Actin or β-tubulin was used as a loading control. (C) DVL1 protein levels in variant DVL1-expressing tissue relative to control tissue. Crosses were performed at 25°C or 29°C. n=3 or 4 independent biological replicates per genotype. Actin or β-tubulin were used as a loading controls. Error bars = mean ± s.d. For Panel A and B, One-way ANOVA and Tukey's post-hoc test was used to compare genotypes. For Panel C Two-way ANOVA and Tukey's post-hoc test was used to compare genotypes and temperatures ** = p<0.01, ****=0.0001.

Disease Models & Mechanisms • Supplementary information

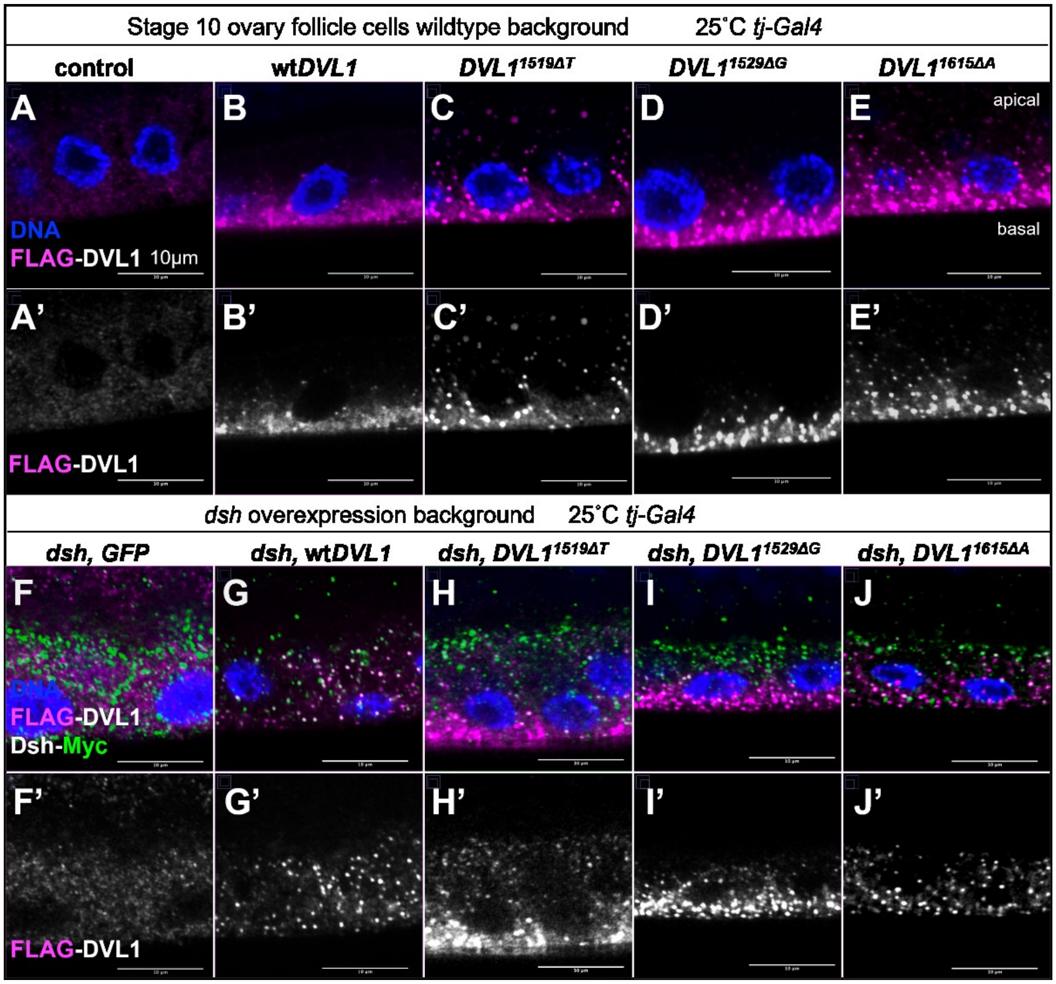

Fig. S9. DVL1 transgene subcellular localization.

(A-E') Single focal plane images showing FLAG-tagged DVL1 protein (magenta) and DNA (blue) staining in (A, A') control (tj-Gal4>w1118) and (B-E') DVL1-expressing tj>wtDVL1, tj>DVL11519ΔT, tj>DVL11529ΔG and tj>DVL11615ΔA) stage 10 ovary follicle cells with corresponding single channel FLAG (white) staining shown in (A'-E'). Crosses were performed at 25°C. (F-J') FLAG (magenta), Myc (green) and DNA (blue) staining in control (F, F') and DVL1-expressing stage 10 ovary follicle cells in a dsh overexpression background (G-J') with corresponding single channel FLAG (white) staining shown in (G'-J'). Images are representative of n=3-10 ovaries per genotype across 2 or 3 independent biological replicates. Crosses were performed at 25°C.

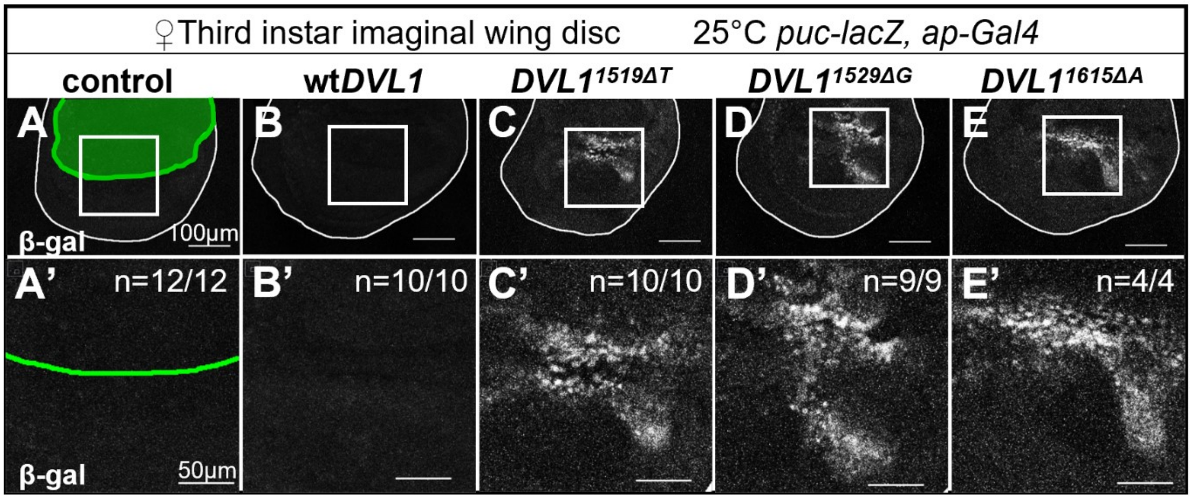

Fig. S10. DVL1 variants induce ectopic JNK signaling.

(**A-E**) Z-stack maximum projection of imaginal wing discs showing β-gal (white) and UAS-transgene expression domain (green) staining in control (**A**) and *DVL1*-expressing female wing discs (**B-E**). (**A'-E'**) Z-stack maximum projection of inset showing β-gal (white) staining in control (**A'**) and *DVL1*-expressing female wing discs (**B'-E'**). n numbers in bottom panels depict the number of wing discs that displayed the phenotype shown in the representative image. Crosses were performed at 25°C and 4-12 female larvae were imaged per genotype across n=2 independent experiments.

Disease Models & Mechanisms • Supplementary information

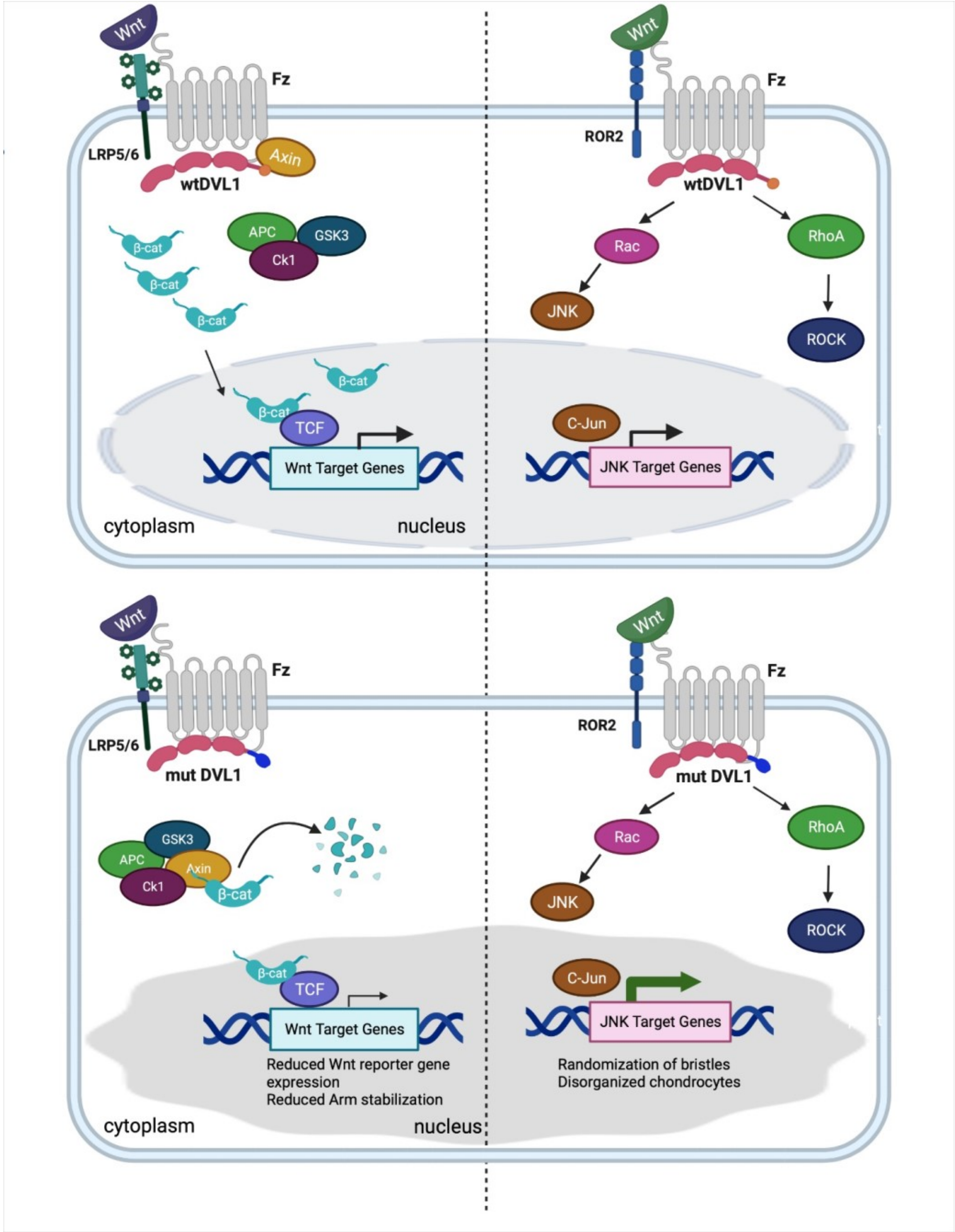

Fig. S11. Summary of signaling impacts that differ between wtDVL1 and mutant DVL1.

Upper panel: In cases of overexpression of human wt*DVL1* the canonical and JNK-PCP pathways are induced. Mild phenotypes are produced such as limb shortening and increased diameter of the cartilage elements. In Drosophila there was mild randomization of wing bristles. Lower panel: In contrast the mutant *DVL1* constructs, caused chondrocyte disorganization, and randomization of bristles. Nuclear shape changes occurred in the chondrocytes expressing variant *DVL1*. In Drosophila mutant *DVL1* strongly repressed Armadillo and increased JNK activity in vivo. In the adult wing, the variants induced novel phenotypes but the underlying pathways are not known.

Table S1. Qualitative analysis of DVL1 forelimb phenotype injected at stage 15 or 20 and fixed at stage 38. Biological replicates and detailed phenotype analysis of embryos injected with viruses into prospective limb region. Limbs can be both short/wide and can have a phenotype affecting the skeletal elements. Abnormalities were mainly bone shortening and thickening.

A. Injected at stage 15

**Phenotypes**	***GFP* (N=24)**	**wt*DVL1* (n=23)**	***DVL1 1519ΔT* (N=11)**	***DVL1 1529ΔG* (N=30)**	***DVL1 1615ΔA* (N=17)**
**Normal**	24<br>100.00%	7<br>30%	5<br>46%	11<br>37%	7<br>41%
**Specimens with a skeletal phenotype**	0	16<br>70%	6<br>55%	19<br>63%	10<br>59%
**Short or wide bones**	0	14<br>61%	6<br>55%	19<br>63%	10<br>59%

B. Injected at stage 20

	***GFP* (N=7)**	**wt*DVL1* (n=19)**	***DVL1 1519ΔT* (N=18)**
**Normal**	7	8	12
**Specimens with a skeletal phenotype**	0	11<br>58%	6<br>50%
**Only humerus affected**	0	5<br>26%	3<br>17%

Disease Models & Mechanisms • Supplementary information

Table S2. Viral spread as determined by GAG staining throughout the developing cartilage in stage 29 and 34 limbs. All embryos were injected at stage 15 and fixed at either stage 29 and 34. Those with strong GAG staining were used in subsequent proliferation and immunostaining experiments.

Stage of fixation	Virus insert	Strong GAG	Uneven GAG	No GAG
29	***GFP*** **(n=5)**	5<br>100.0%	0	0
	**wt*DVL1*** **(n=6)**	6<br>100.0%	0	0
	DVL1 $^{1519\Delta T}$ **(n=8)**	6<br>75.0%	0	2<br>25.0%
	DVL1 $^{1529\Delta G}$ **(n=7)**	5<br>71.4%	1<br>14.3%	1<br>14.3%
	DVL1 $^{1615\Delta A}$ **(n=6)**	5<br>83.3%	1<br>16.7%	0
	**Total (n=32)**	27/32	2/32	3/32
34	***GFP*** **(n=7)**	4<br>57.1%	1<br>14.3%	2<br>28.6%
	**wt*DVL1*** **(n=7)**	6<br>85.7%	1<br>14.3%	0
	DVL1 $^{1519\Delta T}$ **(n=7)**	4<br>57.1%	0	3<br>42.9%
	DVL1 $^{1529\Delta G}$ **(n=7)**	3<br>42.9%	1<br>14.3%	3<br>42.9%
	DVL1 $^{1615\Delta A}$ **(n=7)**	5<br>75.0%	0	2<br>25%
	**Total (n=35)**	22/35	3/35	10/35

Table S3. Qualitative analysis of DVL1 forelimb phenotypes at stage 34. Biological replicates and detailed phenotype analysis and type X collagen expression of embryos injected with viruses into prospective limb region. Limbs can be short, or both short and dysmorphic. Dysmorphic phenotypes were pinching phenotype of the perichondrium. Only specimens with strong GAG expression were analyzed (Table S3).

Stage 34		***GFP*** **(n=4)**	**wt*DVL1*** **(n=6)**	***DVL1 1519ΔT*** **(n=4)**	***DVL1 1529ΔG*** **(n=4)**	***DVL1 1615ΔA*** **(n=5)**
**Type X collagen**	**No expression**	0	5<br>83.3%	0	0	0
	**Uneven Expression**	0	1<br>16.7%	4<br>100%	4<br>100%	5<br>100%
	**Strong Expression**	4<br>100.0%	0	0	0	0
**Phenotype**	**Smooth cartilage rods**	4<br>100.0%	6<br>100.0%	2<br>33.3%	0	0
	**Dysplastic cartilage morphology**	0	0	4<br>66.7%	4<br>100%	5<br>100%

Disease Models & Mechanisms • Supplementary information

Table S4. Qualitative analysis of *DVL1* forelimb phenotype at stage 29. Biological replicates and detailed phenotype analysis of embryos injected with viruses into prospective limb region. Limbs were short, or both short and dysmorphic. Dysmorphic phenotypes were disrupted perichondrium and/or pockets of SOX9-negative tissue within the cartilage as seen in Fig 2. Only specimens with strong GAG expression were analyzed (Table S3).

Stage 29 phenotypes	*GFP* (n=5)	wtDVL1 (n=6)	*DVL1 1519ΔT* (n=6)	*DVL1 1529*ΔG (n=5)	*DVL1 1615*ΔA (n=5)
**Normal**	5	1	2	0	0
**Slightly dysmorphic**	0	2	1	2	2
**Very dysmorphic**	0	0	0	3	2
**short**	0	3	3	0	1

Table S5. Antibodies used in this study

Primary antibody and source	Dilution (all overnight, 4°C)	Antigen Retrieval (steam 15 min 95°C)	Permeabilization/ Pre-treatment	Block goat serum Sigma #G9023	Secondary antibody 1h, 22°C
BrdU DSHB, G3G4	1:20	Diva Decloaker 1:10		10% GS, 0.1% tween-20/PBS, 1h, 22°C	Invitrogen, anti-mouse A11029 1:200
GAG DSHB, AMV-3C2	1:4	10 mM sodium citrate		10% GS, 0.5% tween-20/PBS, 1h, 22°C	Invitrogen, anti-mouse A11029 1:200
Collagen X DSHB, #X-AC9-c	1:250	Diva Decloaker 1:10 Inter Medico, Markham, Ontario, #DV2004MX	0.5% hyaluronidase in Hank's Balanced Salt Solution, 30min (prior to antigen retrieval)	10% GS, 0.5% tween-20/PBS, 1h, 22°C	Invitrogen, anti-mouse A11029 1:200
Golgi antibody BD Labs, GM130, 610822	1:100	Diva Decloaker 1:10	0.5% tween-20, 10 min	10% GS, 0.5% tween-20/PBS, 1h, 22°C	Invitrogen, anti-mouse A10524 1:200
Pan-Prickle Abcam, 15577	1:50	Diva Decloaker 1:10	0.5% tween-20, 10 min	10% GS, 0.5% tween-20/PBS, 1h, 22°C	Invitrogen, anti-rabbit A11034, 1:200
SOX9 Sigma Aldrich HPA001758	1:200	Diva Decloaker 1:10	0.5% Triton-X, 10 min	10% GS, 0.2% TritonX/PBS, 1h, 22°C	Invitrogen, anti-rabbit A10523, 1:200
anti-Flag, Thermo Scientific PA1-9848	1:200			10% GS, 0.2% TritonX/PBS, 1h, 22°C	Invitrogen, anti-rabbit A11034, 1:200
Counterstain	Hoechst 10 µg/ml #33258, Sigma. Prolong Gold antifade (Life Technologies #P36930)				

Disease Models & Mechanisms • Supplementary information

Table S6. CLUSTAL multiple sequence alignment comparing C-terminal peptides in DVL1 and DVL3 variants

A) Alignment of two variants of DVL3 that cause a frameshift and abnormal C-terminus

```
hDVL3_1585_del      GGRRRTRRPGTPSPGAAAANRTTPHAAACGGRGSGRPASAQGRRPASTATAATIPWPAAFAATTHTRATVLPECPLSTAPPC*83

hDVL3_1751-1754_del
GGRRRTRRPGTPSPGAAAANRTTPHAAACGGRGSGRPASAQGRRPASTATAATIPWPAAFAATTHTRATVLPECPLSTAPPC*83
```

B) Alignment of the C-terminal peptides of three DVL1 variants and two DVL3 variants

```
hDVL1_1519del_495-1944   PLPHPAAPGLWVRATPTSTRDPHPASRLPTRTRALAMAAAAPGVSRVKGAKAVGPPGAAA  60
hDVL1_1529del_501-1944     PHPAAPWPLVRATPTSTRDPHPASRLPTRTRALAMAAAAPGVSRVKGAKAVGPPGAAA  58
hDVL1_1615del_501-1944     PHPAAPWPLGQGYPYQYPGPPPCFPPAYQDPGFSYGSGAPGVSRVKGAKAVGPPGAAA  58
hDVL3_1585_del             -----------GGRRRTRRP---------------GTPSPGAA---AANRTTPHAAAC  29
hDVL3_1751-1754_del        -----------GGRRRTRRP---------------GTPSPGAA---AANRTTPHAAAC  29
                                      : ...::.*               :..:**::   :*:...*.:**.

hDVL1_1519del_495-1944   GPRAVRRSVGRRELGAVAVNRITRHRVGWGAAGESVRPASSAVAAAHAVRPRLPPRGSPR  120
hDVL1_1529del_501-1944   GPRAVRRSVGRRELGAVAVNRITRHRVGWGAAGESVRPASSAVAAAHAVRPRLPPRGSPR  118
hDVL1_1615del_501-1944   GPRAVRRSVGRRELGAVAVNRITRHRVGWGAAGESVRPASSAVAAAHAVRPRLPPRGSPR  118
hDVL3_1585_del           GGRGSGRP----------------------ASAQGRRPASTATAATI------PWPAAFA  61
hDVL3_1751-1754_del      GGRGSGRP----------------------ASAQGRRPASTATAATI------PWPAAFA  61
                         *.*:..*:                      *::::.****:*.**:.      *..::..

hDVL1_1519del_495-1944   PTPRPRPIQWWGGHPGDPLSGSWLPSPRN  149
hDVL1_1529del_501-1944   PTPRPRPIQWWGGHPGDPLSGSWLPSPRN  147
hDVL1_1615del_501-1944   PTPRPRPIQWWGGHPGDPLSGSWLPSPRN  147
hDVL3_1585_del           ATTHTRATVL----PECPLS----TAPPC  82
hDVL3_1751-1754_del      ATTHTRATVL----PECPLS----TAPPC  82
                         :*:::*::..    *:.***    ::*..
```

Disease Models & Mechanisms • Supplementary information
